# Genetic Basis of a Cognitive Complexity Metric

**DOI:** 10.1371/journal.pone.0123886

**Published:** 2015-04-10

**Authors:** Narelle K. Hansell, Graeme S. Halford, Glenda Andrews, David H. K. Shum, Sarah E. Harris, Gail Davies, Sanja Franic, Andrea Christoforou, Brendan Zietsch, Jodie Painter, Sarah E. Medland, Erik A. Ehli, Gareth E. Davies, Vidar M. Steen, Astri J. Lundervold, Ivar Reinvang, Grant W. Montgomery, Thomas Espeseth, Hilleke E. Hulshoff Pol, John M. Starr, Nicholas G. Martin, Stephanie Le Hellard, Dorret I. Boomsma, Ian J. Deary, Margaret J. Wright

**Affiliations:** 1 Neuroimaging Genetics, QIMR Berghofer Medical Research Institute, Brisbane, Australia; 2 School of Applied Psychology, Griffith University, Mt Gravatt Campus, Brisbane, Australia; 3 Behavioural Basis of Health Program, Griffith Health Institute and School of Applied Psychology, Griffith University, Brisbane, Australia; 4 School of Applied Psychology, Griffith University, Gold Coast Campus, Southport, Australia; 5 Centre for Cognitive Ageing and Cognitive Epidemiology, University of Edinburgh, Edinburgh, United Kingdom; 6 Centre for Genomic and Experimental Medicine, University of Edinburgh, Edinburgh, United Kingdom; 7 Department of Psychology, University of Edinburgh, Edinburgh, United Kingdom; 8 Department of Biological Psychology, Vrije Universiteit, Amsterdam, The Netherlands; 9 K.G. Jebsen Centre for Psychosis Research and the Norwegian Center for Mental Disorders Research (NORMENT), Department of Clinical Science, University of Bergen, Bergen, Norway; 10 Dr Einar Martens Research Group for Biological Psychiatry, Center for Medical Genetics and Molecular Medicine, Haukeland University Hospital, Bergen, Norway; 11 Genetic Epidemiology, QIMR Berghofer Medical Research Institute, Brisbane, Australia; 12 School of Psychology, University of Queensland, St Lucia, Brisbane, Australia; 13 Molecular Genetic Epidemiology, QIMR Berghofer Medical Research Institute, Brisbane, Australia; 14 Quantitative Genetics, QIMR Berghofer Medical Research Institute, Brisbane, Australia; 15 Avera Institute for Human Genetics, Avera McKennan Hospital & University Health Center, Sioux Falls, South Dakota, United States of America; 16 K.G. Jebsen Center for Research on Neuropsychiatric Disorders, University of Bergen, Bergen, Norway; 17 Department of Biological and Medical Psychology, University of Bergen, Bergen, Norway; 18 Center for Research on Aging and Dementia, Haraldsplass Deaconess Hospital, Bergen, Norway; 19 Department of Psychology, University of Oslo, Oslo, Norway; 20 Molecular Epidemiology, QIMR Berghofer Medical Research Institute, Brisbane, Australia; 21 Norwegian Center for Mental Disorders Research (NORMENT) and the K.G. Jebsen Center for Psychosis Research, Division of Mental Health and Addiction, Oslo University Hospital, Oslo, Norway; 22 Brain Center Rudolf Magnus, Department of Psychiatry, University Medical Center Utrecht, Utrecht, The Netherlands; 23 Alzheimer Scotland Dementia Research Centre, University of Edinburgh, Edinburgh, United Kingdom; Scripps Health and The Scripps Research Institute, UNITED STATES

## Abstract

Relational complexity (RC) is a metric reflecting capacity limitation in relational processing. It plays a crucial role in higher cognitive processes and is an endophenotype for several disorders. However, the genetic underpinnings of complex relational processing have not been investigated. Using the classical twin model, we estimated the heritability of RC and genetic overlap with intelligence (IQ), reasoning, and working memory in a twin and sibling sample aged 15-29 years (N = 787). Further, in an exploratory search for genetic loci contributing to RC, we examined associated genetic markers and genes in our Discovery sample and selected loci for replication in four independent samples (ALSPAC, LBC1936, NTR, NCNG), followed by meta-analysis (N>6500) at the single marker level. Twin modelling showed RC is highly heritable (67%), has considerable genetic overlap with IQ (59%), and is a major component of genetic covariation between reasoning and working memory (72%). At the molecular level, we found preliminary support for four single-marker loci (one in the gene *DGKB*), and at a gene-based level for the *NPS* gene, having influence on cognition. These results indicate that genetic sources influencing relational processing are a key component of the genetic architecture of broader cognitive abilities. Further, they suggest a genetic cascade, whereby genetic factors influencing capacity limitation in relational processing have a flow-on effect to more complex cognitive traits, including reasoning and working memory, and ultimately, IQ.

## Introduction

**Fig 1:**
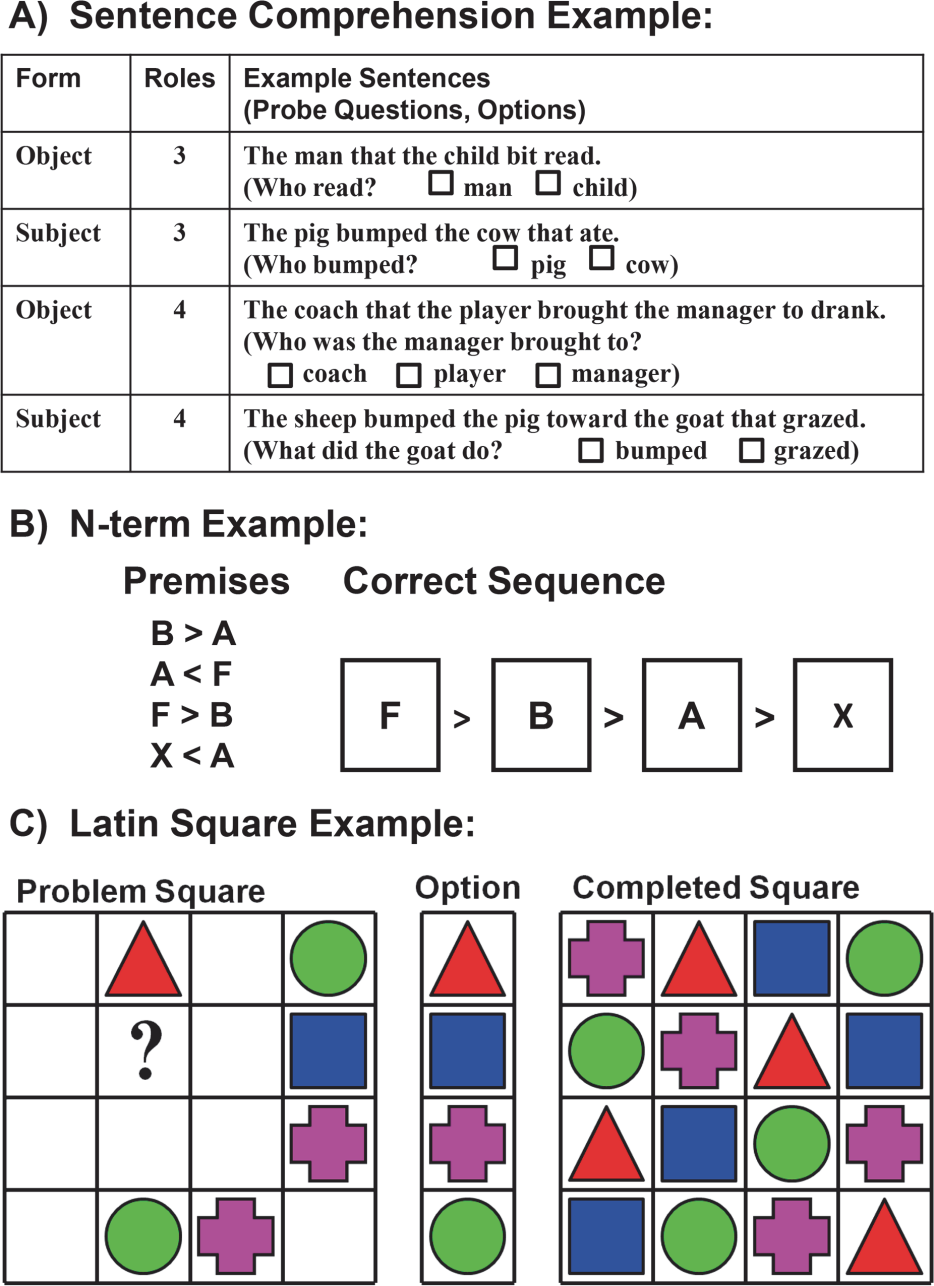
Relational Complexity Tasks. Each task contained items at two or three levels of complexity. The Sentence Comprehension task (A) required processing of noun-verb relations in order to answer a probe question, while the N-term task (B) is an extended version of a transitive inference task, requiring ordering of letters from greatest to smallest based on information given in premises. In the Latin Square task (C) symbols can appear only once in every row or column and participants must solve for a specified cell (marked?). Tasks are described in detail in S1 Text.

Relational processing is defined as the ability to mentally link variables relevant for goal-directed behaviour, and is thought to underlie a diverse range of higher-order cognitive abilities including reasoning, categorisation, planning, quantification, and language [1–12]. One characteristic of relational processing is that it is effortful. It imposes a load on limited cognitive resources and this load increases with the complexity of the relations. Relational complexity (RC) theory [13] quantifies complexity in terms of the RC metric. This metric is domain-general, underlying tasks as divergent as sentence comprehension (understanding multiple “who did what” relations (Fig 1)) and transitive inference (whereby A>C can be inferred from the two relations, A>B and B>C)[14]. The capacity to process complex relational information in order to solve a problem increases from childhood through to young adulthood (most 2 year-olds can process relations between two entities/variables, which increases to three entities/variables for the majority of 5 year-olds, while the relational processing limit for young adults corresponds to four entities related in a single decision [14–16]). This limit on relational processing represents the number of unique entities, or conceptual chunks of information, that can be processed in parallel to arrive at a solution and is proposed to underlie capacity limitations in reasoning (as has been shown for the knight-knave task of suppositional reasoning [16, 17]). Further, it is comparable to the working memory capacity limit of four elements [18]. Indeed, capacity limits in both reasoning and working memory might be based on the limited ability to process complex relational information, which could account for the link found between these traits [19].

Another characteristic of relational processing is its apparent sensitivity to brain abnormalities associated with psychiatric and neurological disorders. Relational processing engages the prefrontal cortex [20, 21], a brain region involved in the integration of information processing that occurs in other specialised brain systems, and that shows a linear pattern of development such that magnitude of activation during tests of executive function increases from childhood through to young adulthood [22–25]. Limits in the ability to process complex relations have recently been associated with increased regional activity within, and functional interactions between, the fronto-parietal and cingulo-opercular control networks, with connectivity between prefrontal regions directly associated with limits in relational processing [12]. Dysfunction of the prefrontal cortex is a central feature of many psychiatric disorders (including schizophrenia, bipolar disorder, attention deficit hyperactivity disorder, and posttraumatic stress disorder [26]) and neurological conditions such as Alzheimer’s disease [27]. Consequently, relational processing ability has been used to characterise executive impairment in Alzheimer’s disease patients [27], and similarly, following stroke [4]. Impaired relational processing is found in schizophrenia [28–30] and patients show altered prefrontal activity during relational processing when compared to controls [31]. This close relationship between cognitive function and psychiatric illness has previously been exploited in the search for genes influencing psychiatric disorders and to gain further insights into the genetic architecture contributing to these disorders [32–34].

Thus, relational processing is identified as a core cognitive trait supporting complex cognitive abilities in healthy individuals [1], and further, is shown to be sensitive to psychiatric and neurological disorder [4, 27, 28]. However, the genetic basis of individual differences in the ability to process relations of varying complexity has not, to our knowledge, previously been examined. Here, using twin and genome-wide analytic approaches, we explore the genetic underpinnings of complex relational processing. Using classical twin modelling and data from a sample of healthy adolescents and young adults (the Discovery sample), we estimated how much of the variance in relational processing was due to genetic factors (i.e. heritability). Based on evidence pointing to the critical role of relational processing in higher cognitive processes [1], we hypothesised that genetic factors influencing relational processing would also be a strong component of general cognitive function, and further, based on the conjecture that capacity limitations in relational processing may reflect a common mechanism restricting both reasoning and working memory [19], that they would account for much of the association found between these two traits. These hypotheses were supported in twin modelling. In exploratory genome-wide analyses of molecular data we then searched for genetic variants (single nucleotide polymorphisms (SNPs)) associated with relational processing. Using a cross-trait consistency approach to reduce noise, we selected a subset of SNPs, which along with our top-ranked SNPs and genes, were assessed for replication in four independent samples. No association results survived correction for multiple testing. However, suggestive results were found for a number of plausible loci.

## Materials and Methods

### Participants

Discovery sample participants were primarily adolescent twins and their singleton siblings from the Cognition Study (N>2700)—a component of the Brisbane Adolescent Twin Study [35]. Sample numbers differed for the twin modelling and genome-wide analyses. Twin modelling was performed on 787 individuals (mean age 17.0±2.2SD years, range 15.9–29.6) for whom measures of relational processing, reasoning, working memory, and IQ were available. These included 138 MZ and 187 DZ twin pairs, 12 triplet trios (one trio included an MZ pair), and 101 single twins or singleton siblings. 752 individuals had data for all four traits. Samples for the genome-wide analyses were restricted by available genotyping (Illumina Human 610-Quad SNP chip [36]), with 497 genotyped individuals (243 families) having relational processing, 481 (234 families) having reasoning, and 483 (234 families) having working memory measures. However, a larger genotyped sample of 1999 individuals (mean age 16.6±1.5 years) from 894 families had measures of IQ. Written, informed consent was obtained from all participants, including a parent or guardian for those aged less than 18 years. The study was approved by the Human Research Ethics Committee at the QIMR Berghofer Medical Research Institute.

### Measures

We used three tasks (Fig 1, S1 Text) across linguistic (Sentence Comprehension) and non-linguistic domains (Latin Square, N-term (a transitive inference task)) to assess relational processing [14, 37, 38]. For each task we assessed participants’ accuracy in processing relations, where successive trials, or blocks of trials within each task, increased in complexity. Using principal component analysis (PCA), we derived a relational complexity (RC) component, which accounted for 63.9% of the variance in the three tasks. Test-retest reliability of RC, assessed in a sub-sample of 20 twin pairs, showed high reliability (0.78; individual tasks ranged 0.44–0.78; Table 1). Full-scale IQ was assessed with the Multidimensional Aptitude Battery (MAB [39]). Reasoning and working memory principal components were each derived from two subtests from the MAB [39] and/or Wechsler Adult Intelligence Scale – Third Edition (WAIS-III [40]) (Table 1). RC was independent of each of the other derived component scores. However, the MAB subtest Arithmetic contributed to both IQ and Reasoning. Details of zygosity determination and genotyping can be found in S1 Table.

**Table 1:** Trait Demographics, Test-Retest Reliability, Phenotypic/*Genetic* Correlations, and Twin Correlations (shown with 95% Confidence Intervals).

	Sentence Comprehension	N-term	Latin Square	Relational Complexity (RC)^a^	Reasoning^a^	Working Memory^a^	IQ^a^
**Trait Demographics**							
N (individuals)	786	785	786	784	755	758	779^b^
Mean±SD	17.1±3.3	11.6±4	2.4±0.4	0±1	0±1	0±1	111.2±12.3
Range	6–22	0–16	0.5–3.0	-4.0 to 1.6	-3.1 to 2.7	-2.5 to 2.8	79–147
**Test-Retest *r*** ^c^	0.74 (0.56–0.84)	0.68 (0.47–0.79)	0.44 (0.13–0.64)	0.78 (0.61–0.86)	-	0.73 (0.58, 0.83)	0.86 (0.81–0.91)
**Phenotypic/*Genetic r*** ^d^							
Sentence Comp	1	*0*.*85 (0*.*67*,*0*.*96)*	*0*.*57 (0*.*28*, *0*.*81)*	*0*.*90 (0*.*81*, *0*.*96)*	*0*.*76 (0*.*55*, *1*.*00)*	*0*.*71 (0*.*52*, *0*.*98)*	*0*.*70 (0*.*56*, *0*.*92)*
N-term	0.56 (0.51–0.61)	1	*0*.*91 (0*.*70*, *1*.*00)*	*0*.*99 (0*.*94*, *1*.*00)*	*0*.*83 (0*.*56*, *1*.*00)*	*0*.*45 (0*.*14*, *0*.*75)*	*0*.*74 (0*.*55*, *0*.*94)*
Latin Square	0.40 (0.34–0.46)	0.47 (0.41–0.52)	1	*0*.*87 (0*.*74*, *0*.*96)*	*0*.*75 (0*.*49*, *0*.*95)*	*0*.*21 (0*.*00*, *0*.*53)*	*0*.*60 (0*.*40*, *0*.*81)*
RC	0.83 (0.80–0.85)	0.82 (0.80–0.84)	0.75 (0.72–0.78)	1	*0*.*84 (0*.*66*, *1*.*00)*	*0*.*52 (0*.*29*, *0*.*79)*	*0*.*75 (0*.*62*, *0*.*92)*
Reasoning	0.49 (0.43–0.55)	0.51 (0.46–0.57)	0.46 (0.40–0.52)	0.61 (0.56–0.66)	1	*0*.*70 (0*.*45*, *0*.*90*	*0*.*86 (0*.*77*, *0*.*99)*
Working Memory	0.49 (0.43–0.55)	0.37 (0.30–0.44)	0.27 (0.20–0.34)	0.48 (0.41–0.53)	0.52 (0.46–0.57)	1	*0*.*56 (0*.*39*, *0*.*79)*
IQ	0.56 (0.51–0.61)	0.56 (0.50–0.60)	0.47 (0.41–0.52)	0.65 (0.61–0.69)	0.75 (0.71–0.78)	0.49 (0.43–0.55)	1
**Twin *r*** ^e^							
MZ Pairs	0.54 (0.42, 0.64)	0.48 (0.34, 0.58)	0.45 (0.31, 0.56)	0.67 (0.58, 0.74)	0.62 (0.52, 0.70)	0.63 (0.53, 0.71)	0.83 (0.81, 0.85)
DZ Pairs	0.30 (0.19, 0.40)	0.32 (0.20, 0.41)	0.20 (0.08, 0.30)	0.37 (0.27, 0.46)	0.39 (0.28, 0.48)	0.38 (0.26, 0.47)	0.42 (0.37, 0.47)

^a^ **RC** was derived from principal components analysis (PCA) of the Sentence Comprehension, N-term, and Latin Square tasks and accounted for 63.9% of variance. **Reasoning**, accounting for 70.2% of the variance in PCA, was derived from the Matrix Reasoning and Arithmetic subtests from the WAIS-III [40] and MAB [39] respectively (note that Arithmetic is contributing to both IQ and Reasoning). **Working Memory,** accounting for 79.1% variance in PCA, was derived from Digit Span Backwards and Letter Number Sequencing (WAIS-III [40]). **IQ** was derived from scaled scores from three verbal (Information, Arithmetic, Vocabulary) and two performance subtests (Spatial, Object Assembly) from the MAB [39].

^b^ For genome-wide association, a larger sample (1999) was used.

^c^ Based on 20 pairs retested for RC and Working Memory (mean interval = 3.3±1.6 months) and an independent set of 50 pairs retested for IQ (mean interval = 3.4±1.0 months) [85]. Test-retest for Reasoning could not be computed due to non-overlap of retest samples for the contributing variables. Note that reliability for Latin Square increased to 0.60 if three individuals showing substantial improvement were dropped.

^d^ Genetic correlations were derived from Cholesky decomposition that allowed for additive genetic, common environmental, and unique environmental influences.

^e^ MZ = monozygotic, DZ = dizygotic. Note that twins were paired with a non-twin sibling where possible to create additional pseudo-DZ pairs. For all variables, correlations between same-sex co-twins could be collapsed over sex for MZ and DZ pairs (i.e. MZ male and female pairs, DZ male and female pairs, Δχ^2^_1_ ranged 0.0–2.5) indicating that the magnitude of genetic and environmental influences did not differ significantly between males and females. Further, indicating that sources of influence do not differ significantly between males and females, the opposite-sex correlations could be set equal to the same-sex DZ correlations for all variables (Δχ^2^_1_ ranged 0.6–3.3), with the exception of IQ (Δχ^2^_1_ = 4.2). This suggests that for IQ there may not be complete overlap in genetic sources of influence for males and females.

### Twin Modelling – Discovery Sample

Classical twin models were employed to estimate heritability and to explore genetic covariation (i) among the three relational processing tasks, (ii) between RC and IQ, and (iii) to assess the degree to which sources influencing RC also contribute to the covariation between reasoning and working memory. This method does not use the genotype data, but rather, utilizes the genetic relationship between twins. Monozygotic (MZ) twins share 100% of their genetic material, while dizygotic (DZ) twins and non-twin siblings share on average 50% of their genetic material.

Twin modeling was performed at univariate and multivariate levels using the structural equation software package Mx [41]. Variance due to individual differences was decomposed into additive genetic (A), common environmental (C), and unshared environmental (E) sources, and multivariate models provided variance/covariance matrices from which genetic and environmental correlations were calculated. We assessed the fit of a series of models, including independent and common pathway models and/or Cholesky decomposition [41] to determine which pattern of covariation best fitted the data.

Prior to modeling, the relational processing measures were transformed (log or square root, S1 Table (distributions for the RC component are also shown in S1 Table)) and all measures were standardized (z-scores, *M* = 0±1). We found no consistent birth-order, zygosity, or age effects. Males had slightly, but significantly, higher IQ and reasoning scores than females, so sex was included as a covariate. No sex effects were found for the relational processing measures or working memory (S2 Table).

### Genome-wide Analyses

#### Discovery Sample

Exploratory genome-wide association (GWA) and gene-based tests were conducted to identify loci influencing RC. To reduce noise, we compared these results to those for reasoning, working memory, and IQ—traits shown in the twin modeling to have a substantial genetic overlap with relational processing and as relational processing is theorized to play a crucial role in each [1, 19]. Only associations found to be consistent across traits, in addition to top hits, were taken forward for replication.

Individual SNPs were tested for association with the family-based SCORE test implemented in the software program Merlin [42]. Merlin accounts for the relatedness of individuals, including MZ twins. Sex, age, and population stratification effects (i.e., the first 3 multi-dimensional scaling scores for each individual from a stratification analysis) were included as covariates. Of the top 50 SNPs associated with RC (where SNPs were in high linkage disequilibrium (≥0.5, identified using SNAP [43]), only one was retained), those with p-values less than 0.05 for all three additional traits were chosen for replication. As our IQ sample was four times that for relational processing, we repeated this process with the top 50 IQ SNPs (i.e., selecting if p<0.05 for RC, reasoning, and working memory). From these 100 SNPs, 10 showed consistency across trait, and including the top hit for IQ (included due to larger sample), a total of 11 SNPs were selected for replication. The software ANNOVAR [44] was used to identify those SNPs in or near genes (build version: hg18).

In addition, to determine if any genes had an excess of SNPs with small p-values, the GWA results were examined in gene-based analyses performed using VEGAS [45], a versatile gene-based association test that is suitable for family-based GWA. It assigns SNPs to autosomal genes, with gene boundaries of ±50kb, and takes into account gene length and linkage disequilibrium. The best performing genes for RC and IQ were selected for replication. GWA and gene-based significance levels, after adjusting for multiple testing and two correlated traits, were 3.1x10^-8^ and 1.7x10^-6^ respectively (S1 Table).

#### Replication and Meta-Analysis

Using four independent samples previously described—*Avon Longitudinal Study of Parents and Children* (ALSPAC [46], N = 4078), *Lothian Birth Cohort 1936* (LBC1936 [47, 48], N = 1005), *Netherlands Twin Registry* (NTR [49, 50], N = 920), and *Norwegian Cognitive NeuroGenetics* (NCNG [51], N = 670)—we attempted to replicate associations for the 11 SNPs and two genes. While none of the independent groups had measures specifically designed to quantify complex relational processing, all had measures of reasoning, working memory, and/or IQ (to which relational processing is proposed to contribute [1]) that could be used as proxies. A full description of these data and cohort-specific association and gene-based analyses is given in S3 Table.

We extracted summary statistics for the 11 markers for reasoning, working memory, and IQ (available for four, two, and three replication samples respectively), which together with the Discovery sample, were meta-analysed in METAL [52] using p-values across studies and with sample size and direction of effect taken into account. As the meta p-value significance may be slightly inflated with related individuals we used family number for sample size for the Australian (Discovery) and Dutch samples.

## Results

### Twin Modelling – Discovery Sample

Mean performance, reliability, and correlations (phenotypic (*r*_*p*_), genetic (*r*_*g*_), and twin) are shown in Table 1. Performance was moderately correlated between the three relational complexity tasks (*r*_*p*_ = 0.40–0.56, *r*_*g*_ *=* 0.57–0.91) and with IQ (*r*_*p*_ = 0.47–0.56, *r*_*g*_ *=* 0.60–0.74), with genetic correlations being stronger than phenotypic. Similarly, RC was strongly correlated with IQ (0.65 (*r*_*g*_ = 0.75)), as well as reasoning (0.61 (*r*_*g*_ = 0.84)), and working memory (0.48 (*r*_*g*_ = 0.52)).

**Fig 2:**
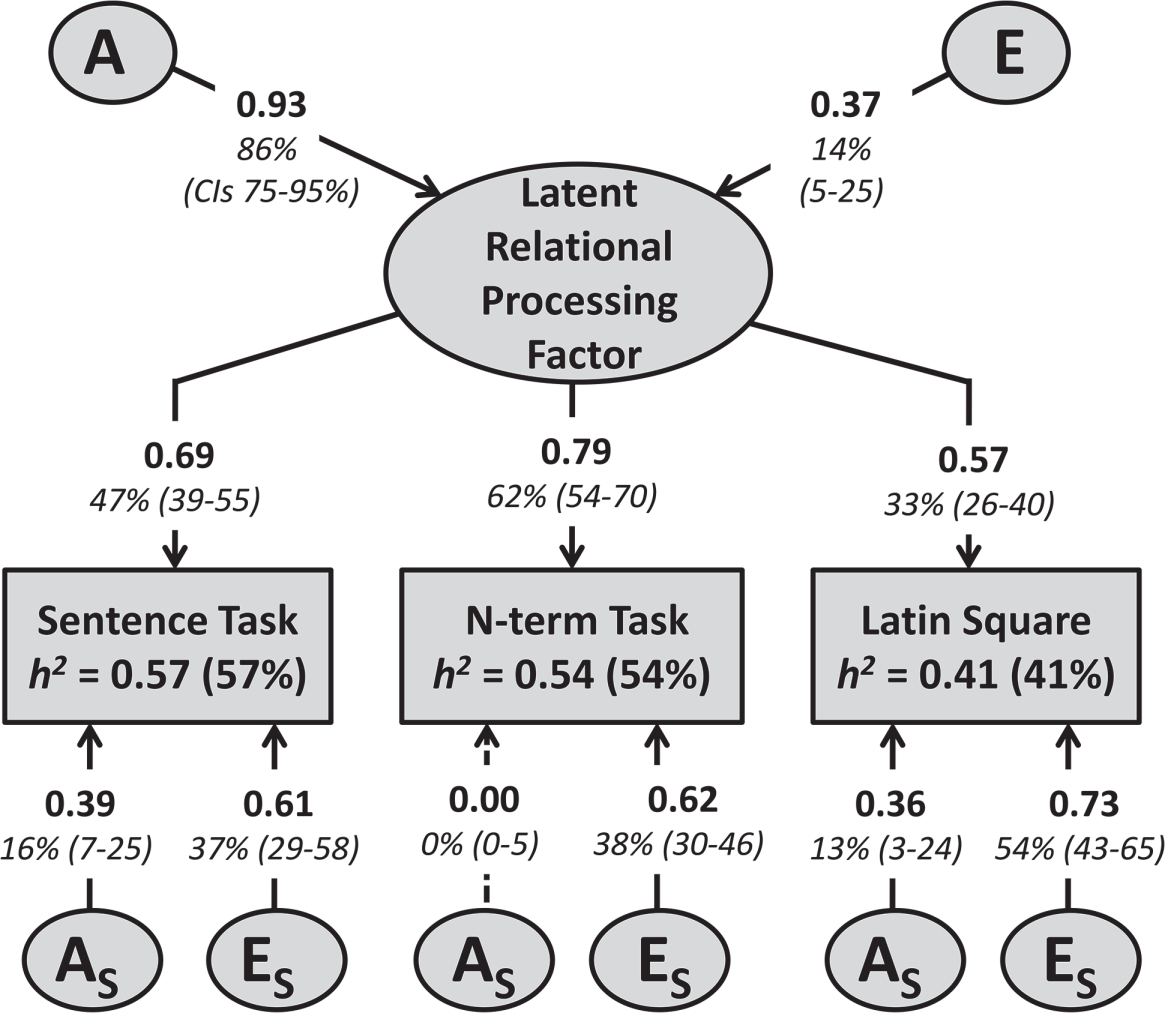
Strongly Genetic Latent Factor Influences Individual Relational Complexity Tasks. In this common pathway model [41], the latent factor is influenced by additive genetic (A) and non-shared environmental (E) sources. Remaining variance was accounted for by genetic and environmental influences specific to each task (A_s_ and E_s_). Dashed lines indicate non-significant pathways. Heritability (*h^2^*) is indicated for each task. For greater detail see S2 Text and for multivariate model-fitting see S5 Table.

**Fig 3:**
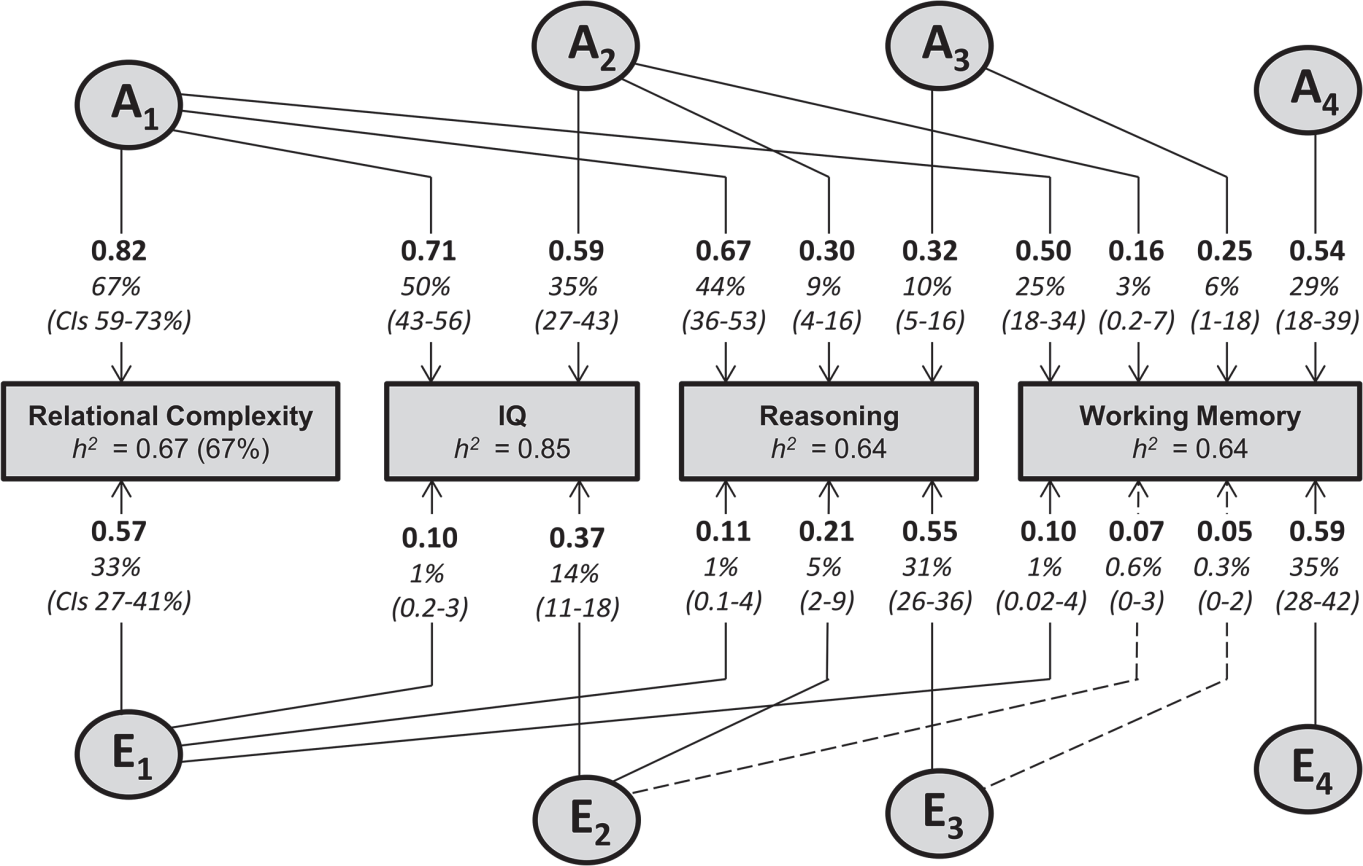
Genetic Sources Influencing Relational Complexity (RC) Underpin Intelligence and Covariation Between Reasoning and Working Memory. In this Cholesky decomposition [41], additive genetic factors are designated A_1_-A_4_, and non-shared environmental factors E_1_-E_4_ (dashed lines indicate non-significant pathways). Heritability (*h^2^*) is shown for each trait. Parameter estimates are standardised such that when squared they indicate the percentage of variance accounted for (shown with 95% confidence intervals). Variable order was chosen to examine (i) the contribution of sources influencing RC (i.e. A_1_, E_1_) to the covariation between reasoning and working memory, and (ii) if sources influencing IQ added to this covariation independently of RC. For greater detail see S2 Text and S1 Fig (focussing on covariation between RC and IQ) and S2 (showing alternative variable orders for the quadrivariate Cholesky). Note that unless there are qualitative sex differences, the order of traits in a Cholesky decomposition does not change measure of fit (or conclusion).

Univariate model-fitting showed that common environmental influences could be dropped without loss of fit for all traits (S4 Table). However, it should be noted that if there are small but true common environmental influences, these may bias heritability estimates upwards. Heritability (i.e. *h* ^*2*^) estimates for the individual relational complexity tasks were in the moderate range (41–57%, Fig 2, see also S2 Text and S5 Table). RC was slightly more heritable (67%), and of similar magnitude to the reasoning and working memory factors (both 64%), though less heritable than IQ (85%, Fig 3). Heritability of a latent relational processing factor, derived from common pathway modelling of the individual relational processing tasks, was higher (86%, Fig 2) as measurement error and environmental influences specific to each task were partialled out. The latent relational processing factor accounted for 33–62% of variation in the individual relational processing tasks, and shows that a common genetic source is a strong influence on performance in all tasks.

Consistent with our hypotheses, RC was a strong component of IQ (Fig 3, S2 Text) with genes accounting for most (91%) of the association (i.e. *r*_*p*_ = 0.65). Even so, RC was not totally subsumed within IQ with 40% of its genetic variance being specific (S1 Fig). The genetic source influencing RC (i.e. A1) also accounted for 69% of genetic variation in reasoning and 39% of genetic variation in working memory. Importantly, factors influencing RC accounted for 67% of the total covariation (72% of genetic covariation) between reasoning and working memory. Independent of RC, IQ accounted for an additional 12% of the total covariation (10% of genetic covariation), while processes independent of both RC and IQ accounted for the remaining 21%. Taking into account the genetic overlap between RC and IQ, RC accounted for 8% of the genetic covariation independently of IQ (S2 Fig shows examples of alternative variable order).

### GWA Analyses

#### Discovery Sample

No associations for either RC or IQ reached genome-wide significance (i.e., 3.1x10^-8^; for quantile-quantile plots see S3 Fig). For RC, the strongest association was with rs4390263, *p* = 1.4x10^-6^. This SNP was also suggestive (*p*<0.05) for the related traits of reasoning, working memory, and IQ. In addition, a further five of the top 50 RC SNPs were suggestive across all three additional cognitive traits (S6 Table). For IQ, the strongest association was with rs1242923 (*p* = 5.0x10^-6^). In addition, four of the top 50 IQ SNPs were suggestive for RC, reasoning, and working memory (S7 Table). These 11 SNPs, shown in Table 2, were taken forward for replication. Minor allele frequencies for all samples are shown in S8 Table.

**Table 2:** Association for SNPs Carried Forward to Replication: Discovery/Replication Samples and Meta-Analyses.

		Australian Discovery		English ALSPAC		Scottish LBC1936		Dutch NTR		Norwegian NCNG		Meta-Analyses	
SNP	Gene, Location	Effect (SE)	P value	Effect (SE)	P value	Effect (SE)	P value	Effect (SE)	P value	Effect (SE)	P value	Z-score	P value
**1. Relational Complexity (RC) or Reasoning** ^**b**^ **:**													
rs10209999	Intergenic, 2:138312920	-0.30 (0.08)	***1*.*4x10*** ^***-4***^	-0.01 (0.03)	0.584	-0.01 (0.03)	0.884	-	-	-0.52 (0.26)	***0*.*045***	-1.943	**0.052**
rs2442756	*VPS13B*, 8:99816910	0.28 (0.07)	***1*.*5x10*** ^***-4***^	0.02 (0.02)	0.354	0.01 (0.03)	0.861	-0.08 (0.05)	0.162	-0.55 (0.23)	***0*.*020***	0.472	0.637
rs11195283	*RBM20*, 10:110721690	-0.28 (0.07)	***1*.*4x10*** ^***-4***^	0.00 (0.02)	0.888	-0.01 (0.03)	0.806	0.08 (0.05)	0.144	0.40 (0.24)	**0.096**	-0.071	0.943
rs4390263^c^	near *NPS*,10:127556291	-0.35 (0.07)	***1*.*4x10*** ^***-6***^	0.00 (0.02)	0.877	0.08 (0.03)	***9*.*5x10*** ^***-3***^	0.04 (0.05)	0.400	0.39 (0.22)	**0.081**	0.991	0.322
rs12882037	near ESRRB, 14:75350842	-0.36 (0.09)	***3*.*7x10*** ^***-5***^	0.02 (0.03)	0.491	-0.01 (0.03)	0.780	0.10 (0.07)	0.150	-0.30 (0.24)	0.225	-0.419	0.676
rs3827183	*DOPEY2*, 21:36289107	-0.41 (0.11)	***1*.*2x10*** ^***-4***^	-0.02 (0.03)	0.630	0.01 (0.03)	0.750	-0.09 (0.08)	0.252	0.55 (0.36)	0.128	-0.812	0.417
**2. IQ or Equivalent** ^c^ **:**													
rs2964546	Intergenic, 5:115407800	0.15 (0.04)	***6*.*7x10*** ^***-5***^	0.04 (0.02)	0.139	-0.03 (0.03)	0.402	-	-	0.62 (0.64)	0.346	2.613	***9*.*0x10*** ^***-3***^
rs7801010	*DGKB*, 7:14275141	0.16 (0.08)	***4*.*5x10*** ^***-5***^	0.00 (0.03)	0.941	0.06 (0.03)	**0.060**	-	-	-0.12 (0.64)	0.851	2.135	***0*.*033***
rs12419146	*PRR5L*, 11:36331921	0.37 (0.09)	***3*.*0x10*** ^***-5***^	0.00 (0.06)	0.993	0.07 (0.03)	***0*.*041***	-	-	1.03 (1.74)	0.554	1.900	**0.057**
rs1242923	*ABHD4*, 14:22605603	-0.17 (0.04)	***4*.*8x10*** ^***-6***^	-0.02 (0.02)	0.489	-0.01 (0.03)	0.766	-	-	1.15 (0.59)	**0.051**	-1.739	**0.082**
rs4482248^d^	Intergenic, 15:96755114	-0.18 (0.04)	***1*.*7x10*** ^***-5***^	-0.06 (0.03)	***0*.*021***	0.01 (0.03)	0.776	-	-	0.05 (0.07)	0.943	-3.264	***1*.*1x10*** ^***-3***^

NOTE: P values <0.10 are shown in bold, while those <0.05 are also underlined. Results are reported for the minor allele and are standardised for all cohorts excepting NCNG. Minor allele frequencies are reported in S8 Table. In the Discovery sample gene-based test for RC: *NPS* was the top ranked gene (*p* = 1.5x10^-5^), while *VPS1*

*3B* and *DOPEY2* were nominally associated (*p* = 0.02, 0.04 respectively). In the gene-based test for IQ: *DGKB* and *ABHD4* were nominally associated (*p* = 0.03, 8.1x10^-4^ respectively). *RBM20* and *PRR5L* were not VEGAS-listed genes.

^a^Sample sizes: Australian Discovery (1. N = 497 (243 families); 2. N = 1999 (894 families), English ALSPAC (N = 4078 unrelated), Scottish LBC1936 (N = 1001 unrelated), Dutch NTR (N = 920 (340 families)), Norwegian NCNG (N = 670 unrelated).

^b^This set of SNPs are from the top 50 RC SNPs. Results for all replication SNPs are shown in supplementary S9–S10 Tables. Measures examined in meta-analysis: Discovery – RC; ALSPAC/LBC1936/NCNG – Matrix Reasoning; NTR—Raven’s Progressive Matrices.

^c^This set of SNPs are from the top 50 IQ SNPs. Results for all replication SNPs are shown in supplementary S9–S10 Tables. Measures examined in meta-analysis: Discovery—IQ from the Multidimensional Aptitude Battery (5 subtests), ALSPAC and NCNG – IQ from the WASI (2 subtests—includes Matrix Reasoning), LBC1936—Moray House.

^d^Further support for this SNP was found in meta-analysis for Working Memory (rs4390263, *p* = 0.023; rs4482248, *p* = 0.026; N = 1825; Discovery – PCA-derived Working Memory; LBC1936 – Letter Number Sequence; NCNG – Digit Symbol).

#### Replication and Meta-analysis

Of 109 association tests (11 SNPs for 10 related traits across 4 independent groups (excluding rs10209999 for the NTR cohort), 11 were nominally associated (*p*<0.05, S9–S10 Tables). This exceeds that expected by chance (0.05*109 = 5.5). However, direction of results was not always consistent across groups. Meta-analysis of IQ results from 4 groups (N = 7083) revealed three independent nominally associated SNPs (intergenic SNPs rs2964546, rs4482248, and a SNP in the gene *DGKB*, rs7801010 (Table 2)). Nominal associations were also found for working memory (3 groups, N = 1825) at two loci (rs4390263 (*NPS*) and rs4482248 (intergenic)).

### Gene-based Tests

#### Discovery Sample

No genes reached the significance threshold (1.7x10^-6^). The top ranked gene for RC was *NPS* (*p* = 1.5x10^-5^), consistent with the top GWAS SNP (rs4390263). The top ranked gene for IQ was *FAM105A*, *p* = 3.2x10^-5^. These 2 genes were taken forward for replication.

#### Replication

Tests for *NPS* resulted in suggestive *p*-values (ranging 0.007 to 0.06) for reasoning in three of the four replication cohorts (Table 3). The only exception was the NTR cohort, for which SNP overlap was small (86% of NTR SNPs for *NPS* and *FAM105A* were specific to that cohort). For *FAM105A*, no consistent support was found (Table 3).

**Table 3:** Discovery and Replication Gene-based Test Results: *NPS* and *FAM105A*.

	P values x Cognitive Trait			
	Relational Complexity	IQ	Reasoning	Working Memory
***NPS* (10q26.2)**				
Australian Discovery	***1*.*5x10*** ^***-5***^	0.183	**0.053**	**0.076**
English ALSPAC	**-**	0.239	***2*.*1x10*** ^***-2***^	-
Scottish LBC1936	**-**	0.111	***7*.*4x10*** ^***-3***^	0.343
Dutch NTR^a^	**-**	-	0.582	-
Norwegian NCNG	**-**	0.258	**0.063**	-
***FAM105A* (5p15.2)**				
Australian Discovery	0.162	***2*.*8x10*** ^***-5***^	0.122	0.847
English ALSPAC	-	0.775	0.292	-
Scottish LBC1936	-	0.652	0.185	0.899
Dutch NTR^a^	-	-	0.722	-
Norwegian NCNG	-	**0.063**	0.139	-

NOTE: P values <0.10 are shown in bold, while those <0.05 are also underlined. Sources for IQ, reasoning, and working memory varied between cohorts (**IQ**: Discovery—verbal and performance subtests (5) from the Multidimensional Aptitude Battery (MAB), ALSPAC and NCNG – subtests (2) from the WASI (includes Matrix Reasoning), LBC1936—Moray House; **Reasoning**: Discovery – PCA-derived reasoning (Matrix Reasoning, Arithmetic (MAB subtest)), ALSPAC/LBC1936/NCNG—Matrix Reasoning, NTR—Raven’s Progressive Matrices; **Working Memory**: Discovery – PCA-derived working memory (Digit Span Backwards, Letter Number Sequencing), LBC1936—Letter Number Sequencing.)

^a^Due to differences in genotyping platform Illumina for Discovery, NCNG, LBC1936, ALSPAC; Affymetrix for NTR) SNP overlap

for *NPS* and *FAM102A* SNPs between NTR and the other cohorts was low (86% of NTR SNPs were specific to that cohort).

### Post Hoc Links to Cognition and Related Traits

Of the 11 SNPs selected for replication, eight were located in a gene (rs7801010 (*DGKB*), rs2442756 (*VPS13B*), rs11195283 (*RBM20*), rs12419146 (*PRR5L*), rs1242923 (*ABHD4*), and rs2837183 (*DOPEY2*), or near a gene (rs4390263 (3.62kb downstream of *NPS*); rs12882037 (20.5kb upstream of *ESRRB)*). As outlined in S11 Table, seven of these genes have, to varying degrees, plausible links to cognition (i.e. *DGKB*, *NPS*, *VPS13B*, *RBM20*, *ABHD4*, *ESRRB*, and *DOPEY2*), with some active in systems implicated in schizophrenia pathology (*NPS*, *DGKB*, *ABHD4*, *ESRRB*).

None of the 11 SNPs have been identified in previous GWA meta-analyses of (i) adult cognition (N = 3,511 [53, 54]), (ii) childhood cognition (N = 12,441 [55]), or (iii) educational attainment (N = 126,559 [56]), although suggestive evidence in Norwegian and British samples indicates that the gene *DGKB* may influence fluid intelligence (*p* = 0.04 and 0.001 respectively [54]). For sample overlap with these studies, see S12 Table.

## Discussion

This is the first study to examine the extent of genetic influence on the ability to process complex relational information. Relational processing is known to impose processing loads that increase with the complexity of relational information [14, 15, 57]. Furthermore, individual differences in this ability have been demonstrated [15, 57]. Here, the role of processing complex relations (i.e. RC) is explored as a core component of cognitive function, as a foundation for both reasoning and working memory [1, 19], and as a potentially important endophenotype for psychiatric and neurological disorders [27, 28, 30]. First we show that RC is strongly heritable (i.e., genetic sources account for 67% of individual variability). This heritability estimate is similar to that found here for reasoning and working memory domains (Fig 3) and in other studies for higher-order cognitive functions [58]. Consistent with prior work [1, 19, 57], RC accounted for a substantial amount of the variance in IQ and the majority of covariation between reasoning and working memory. Here we show that these relationships are driven almost entirely by overlapping genetic influences. Further, in exploratory analyses, we searched for common genetic variants that influence RC, with meta-analyses providing suggestive support for four loci.

Our analyses show RC is characterised by substantial individual variation that can be reliably measured. Genetic and environmental influences were independent of sex and a strong genetic source influenced variation in our adolescent and young adult sample. Typically, the heritability of cognitive abilities increases steeply throughout childhood and adolescence to young adulthood, with common (shared) environmental influences becoming less important over the lifespan [59–61]. Heritability then remains relatively stable through middle and old age [62, 63], although decreases in later life have sometimes been indicated [64, 65] and trajectories can also be measure dependent, with for example, heritability of memory performance reported to increase in old age [64, 66]. Further, it has been shown that there is substantial overlap between genetic sources influencing cognitive ability in childhood and old age [67].

The heritability of RC in our adolescent and young adult sample was maximised through computation of a principal component from tests spanning linguistic and non-linguistic domains. An important characteristic of the RC metric is that it defines cognitive complexity in a way that is applicable to different content domains [14]. In this, RC somewhat reflects the extraction of IQ from multiple verbal and performance abilities. To some extent, the higher heritability in a principal component score may reflect the reduction of random noise, as measurement error inflates environmental influence and thereby reduces heritability. Similarly, we found that heritability is further increased when a latent relational processing factor is derived from common pathway modelling of individual relational processing tasks (86% vs. 67%), as uncorrelated measurement error, plus genetic and environmental influences specific to each task, are partialled out of the latent factor. While our results suggest that our core ability to process complex relations is very strongly influenced by our genetic make-up, this does not preclude the importance of environmental effects, which can influence heritability when (a) our response to the environment is partly dependent upon our genotype (gene-environment *interaction*), or (b) our genetically influenced preferences lead us to seek out particular environments (gene-environment *correlation*) [68]. Further, no significant common environmental factor was identified, but it is possible that in larger samples the larger statistical power would allow detection of such influences. We note however, that evidence of shared environmental influences in adults is very limited for measures of cognition. Heritability scores derived from DNA using Genome-wide Complex Trait Analysis (GCTA[69]) show that common genetic variants account for approximately two-thirds of twin study heritability estimates for cognitive abilities, and set a lower bound for such estimates [70].

Previously, we have theorized that relational processing is the foundation of higher cognitive processes [1]. Here we show that genetic sources influencing variability in RC also account for over half of the individual variation in general cognitive ability and for most (91%) of the association between these measures (*r*_*p*_ = 0.65). However, the genetic source influencing RC is not subsumed in that influencing IQ. While there is substantial genetic overlap, a genetic factor independent of IQ accounts for approximately 27% of individual variation in relational processing ability. In contrast, the influence of unique environmental sources is almost entirely specific to each measure.

We have further proposed that the similarity in capacity limitations found for reasoning (i.e. 4 interrelated variables [16]) and working memory (~4 chunks [18]) might be based on the limited ability to form and retain relationships between elements—in other words, a capacity limitation in relational processing [19]. Here we explored the covariation between reasoning and working memory in terms of genetic and environmental sources and the contribution of sources that also influence RC. Reasoning and working memory were moderately correlated (0.52), with genetic sources accounting for the majority (89%) of the covariation (Fig 3). This genetic component of the covariation was substantially influenced (72%) by sources also influencing RC. It also largely reflects that component of general cognitive ability that covaries with relational processing, with RC influencing only 8% of the covariation between reasoning and working memory independently of IQ (and IQ influencing 12% of the covariation independently of RC). This finding is consistent with the perspective that genes influencing variation in the ability to process complex relations thereby also contribute to variability in both reasoning and working memory.

In the present study, while we had substantial power to detect sources of genetic and environmental variance in relational processing using the classical twin design [71], we lacked power for genome-wide association (GWA) due to the complex architecture of traits such as cognition, where many variants of small effect are involved [72]. Thus, our GWA analyses of this novel phenotype are exploratory and our *p*-values are modest. To reduce noise, we used a cross-trait consistency approach and selected eleven SNPs and two genes for replication. This included a total of nine genes (with additional SNPs in intergenic regions), of which most were plausible as candidates for involvement in cognition (S11 Table). Heterogeneity among the cognitive tests across the five cohorts (Australian Discovery, English ALSPAC, Scottish LBC1936, Dutch NTR, and Norwegian NCNG) was unavoidable. Further, our meta-analysis *p*-values did not survive correction for multiple testing and should be considered preliminary. However, in support of the findings, there is converging evidence that the genes they lie in or near could plausibly influence cognitive processes. From our GWA meta-analyses, variants in or near the genes *DGKB* and *NPS*, as well as two intergenic variants (rs4482248 and rs2964546) were implicated. DGKB is a kinase involved in signalling and phospholipid synthesis, which seems to be preponderant in the brain. In humans, DGKB has been associated with stimulating the secretion of insulin [73], a hormone found to have potent effects in the brain, with insulin dysfunction underlying several risk factors implicated in cognitive decline [74]. Recent replicated gene-based association results suggest *DGKB* may influence fluid intelligence [54], while rat studies show DGKB involvement in hippocampal development, with flow-on effects in memory maze tasks [75, 76]. The hippocampus is most commonly known for its involvement in memory processes [77], but it is also involved in relational processing [78]. Similarly, the intergenic SNP rs4482248 may also influence relational processing via the hippocampus, as this SNP has been nominally associated with hippocampal volume in a GWA meta-analysis by the ENIGMA Consortium (N = 21,151) [79]. In addition, both our GWA (rs4390263) and gene-based tests suggest an association between the *NPS* gene and processes related to relational processing. Relational processing is known to be impaired in schizophrenia patients [80, 81] and *NPS* has been implicated in susceptibility for this disorder [82], including a large GWA meta-analysis by the Psychiatric Genomics Consortium (N = 51,695) [83] showing that the minor allele of rs4390263 has a small protective effect. In addition, NPS receptors are reported to modulate verbal memory in schizophrenia patients [82] and central NPS administration has been shown to dose-dependently enhance memory retention in mice [84]. Taken together, these converging lines of evidence are intriguing, but the associations with relational processing reported here should be interpreted cautiously and need replication.

## Conclusions

We find relational processing to be reliable and heritable, and consistent with RC theory [1, 19], capacity limitations for processing complex relations appear to make a substantial contribution to general cognitive ability and to underlie much of the covariation found between reasoning and working memory. Importantly, overlapping genetic sources drive these associations, and as such, genetic factors related to relational processing are identified as an important component of the genetic architecture underlying intelligence. Further, the results are consistent with a genetic cascade effect whereby genetic factors influencing core cognitive traits have flow-on effects to more complex cognitive behaviours. Potentially, genetic sources influencing structural and functional aspects of the prefrontal cortex, a brain region associated with relational processing [12, 20, 21], may be an earlier step in this genetic cascade. Future studies can assess these relationships by including brain imaging measures of prefrontal cortex structure and function in multivariate models similar to those found in the current study and in models examining direction of causation.

## Supporting Information

S1 Fig Bivariate Cholesky Decomposition: RC, IQ. (PDF)

S2 Fig Quadrivariate Cholesky Decomposition: Alternative Variable Orders. (PDF)

S3 Fig Quantile-Quantile Plots. (PDF)

S1 Table Methods: Discovery Sample Genotyping and Preliminary Analyses. (PDF)

S2 Table Results for Assumption Testing and Sex and Age Effects. (PDF)

S3 Table Sample and Analyses Details for the Replication Cohorts. (PDF)

S4 Table Univariate Cholesky Decomposition of Genetic and Environmental Variance: All Traits. (PDF)

S5 Table Multivariate Model-Fitting for Latin Square, N-term, and Sentence Tasks. (PDF)

S6 Table Top 50 Genome-Wide Association Single Nucleotide Polymorphisms (SNPs) for the RC Factor. (PDF)

S7 Table Top 50 Genome-Wide Association Single Nucleotide Polymorphisms (SNPs) for IQ. (PDF)

S8 Table Minor Allele Frequencies. (PDF)

S9 Table Association Results for all Samples (6 Loci Selected from RC Factor Genome-Wide Association). (PDF)

S10 Table Association Results for all Samples (6 Loci Selected from IQ Genome-Wide Association). (PDF)

S11 Table Gene Function. (PDF)

S12 Table Sample Overlap. (PDF)

S1 Text Relational Complexity Task Descriptions. (PDF)

S2 Text Structural Equation Modelling. (PDF)
