# Supplementary material for: Genetic Basis of a Cognitive Complexity Metric
Source: PLoS One. 2015 Apr 10;10(4):e0123886. doi: 10.1371/journal.pone.0123886 (PMC4393228; doi:10.1371/journal.pone.0123886)
Supplement: S1 Fig — (PDF) [file pone.0123886.s001.pdf]

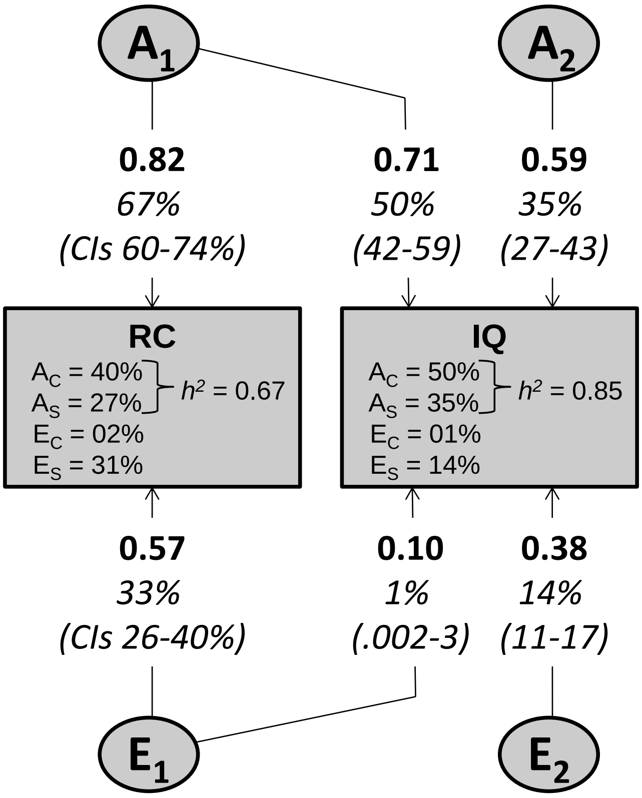

Figure S1. Bivariate Cholesky Decomposition: RC, IQ. This Cholesky decomposition [1] details the genetic and environmental overlap between a Relational Complexity principal component (RC) and IQ.
Cholesky decomposition shows that the RC component was more heritable than the individual RC tasks (0.67 vs. 0.41-0.57), but less heritable than the latent relational processing factor (0.67 vs. 0.86) (Figure 2). IQ was strongly heritable (0.85) and there was considerable genetic overlap between the RC and IQ, such that 60% of genetic variance in RC (40/67) and 59% of the genetic variance in IQ (50/85) is estimated to come from a common genetic source. Even so, this shows that both RC and IQ retain a similar and substantial degree of genetic independence (i.e. 40% for RC (27/67), 41% for IQ (35/85)). In contrast to the genetic overlap almost none was found for unique environmental sources of influence. (As Cholesky decomposition only identifies specific influences for the last variable, we ran two bivariate analyses that allowed both RC and IQ to be the last variable, thus allowing both common (A_C, E_C) and specific (A_S, E_S) influences to be identified for both variables.) Overall, this model shows that genes account for most of the correlation (i.e. 91% (0.82x0.71/((0.82x0.71)+(0.57x0.10))) between RC and IQ.

References

1. Neale MC, Cardon LR (1992) Methodology for genetic studies of twins and families. Dordrecht: Kluwer Academic Publishers.
