# Supplementary material for: Genetic Basis of a Cognitive Complexity Metric
Source: PLoS One. 2015 Apr 10;10(4):e0123886. doi: 10.1371/journal.pone.0123886 (PMC4393228; doi:10.1371/journal.pone.0123886)
Supplement: S2 Fig — (PDF) [file pone.0123886.s002.pdf]

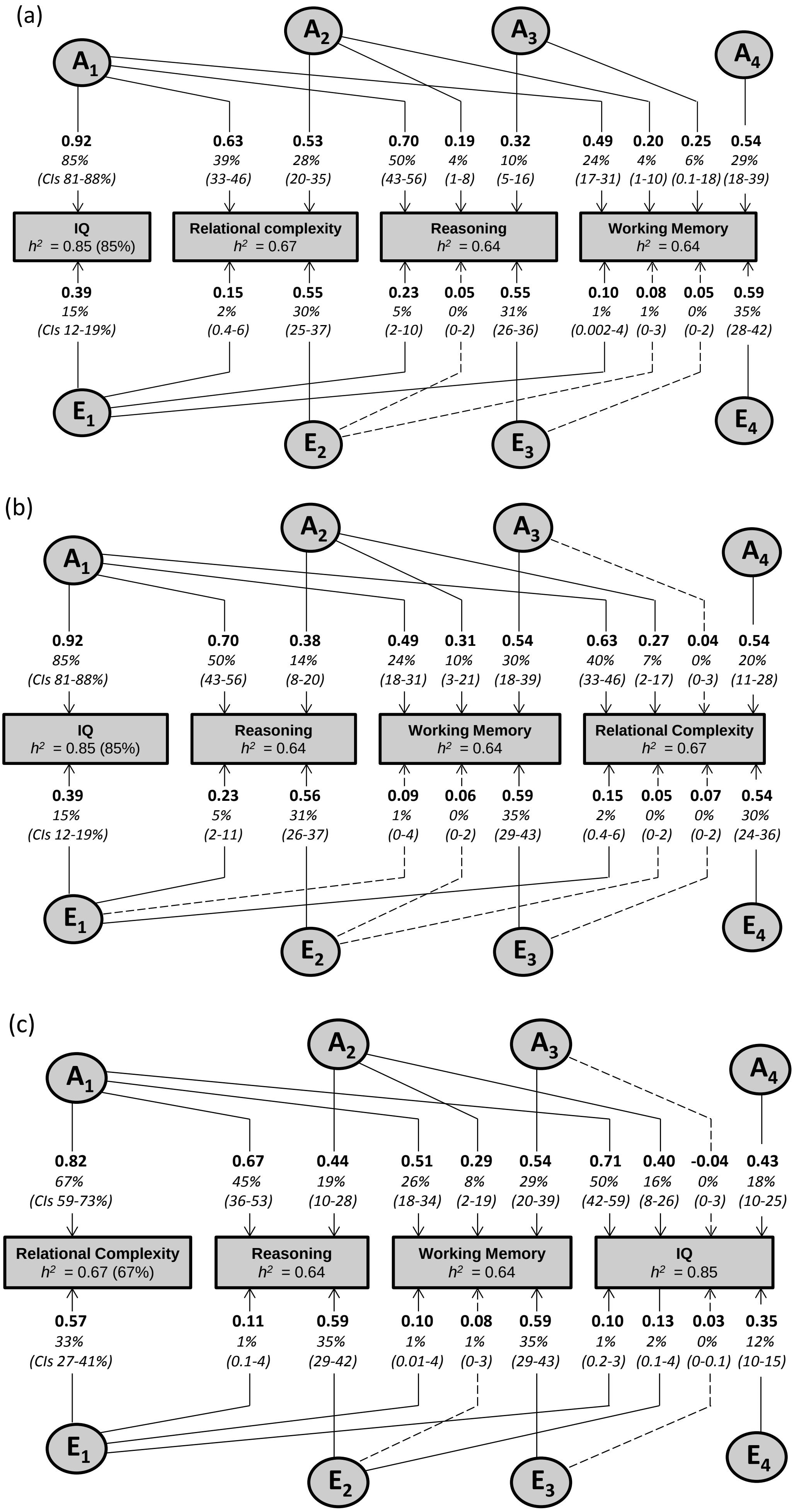

Figure S2. Quadrivariate Cholesky Decomposition: Alternative Variable Orders. These path diagrams show **(a)** reverse order for RC and IQ, **(b)** RC as the last variable, and **(c)** IQ as the last variable. Reversing the order of RC and IQ **(a)** shows that RC accounts for 8% of the covariation between reasoning and working memory independently of IQ (compared to IQ accounting for 12% independently of RC). Having RC and IQ as the last variable shows their genetic independence from the other variables. For RC **(b),** 30% of total genetic variance (20/67) is independent of sources influencing IQ, reasoning, and working memory. For IQ **(c)** 21% of total genetic variance (18/85) is independent of RC, reasoning and working memory. This last estimate is likely artificially reduced due to a common test (Arithmetic) contributing to both reasoning and IQ.
