# Supplementary material for: Genetic Basis of a Cognitive Complexity Metric
Source: PLoS One. 2015 Apr 10;10(4):e0123886. doi: 10.1371/journal.pone.0123886 (PMC4393228; doi:10.1371/journal.pone.0123886)
Supplement: S3 Fig — (PDF) [file pone.0123886.s003.pdf]

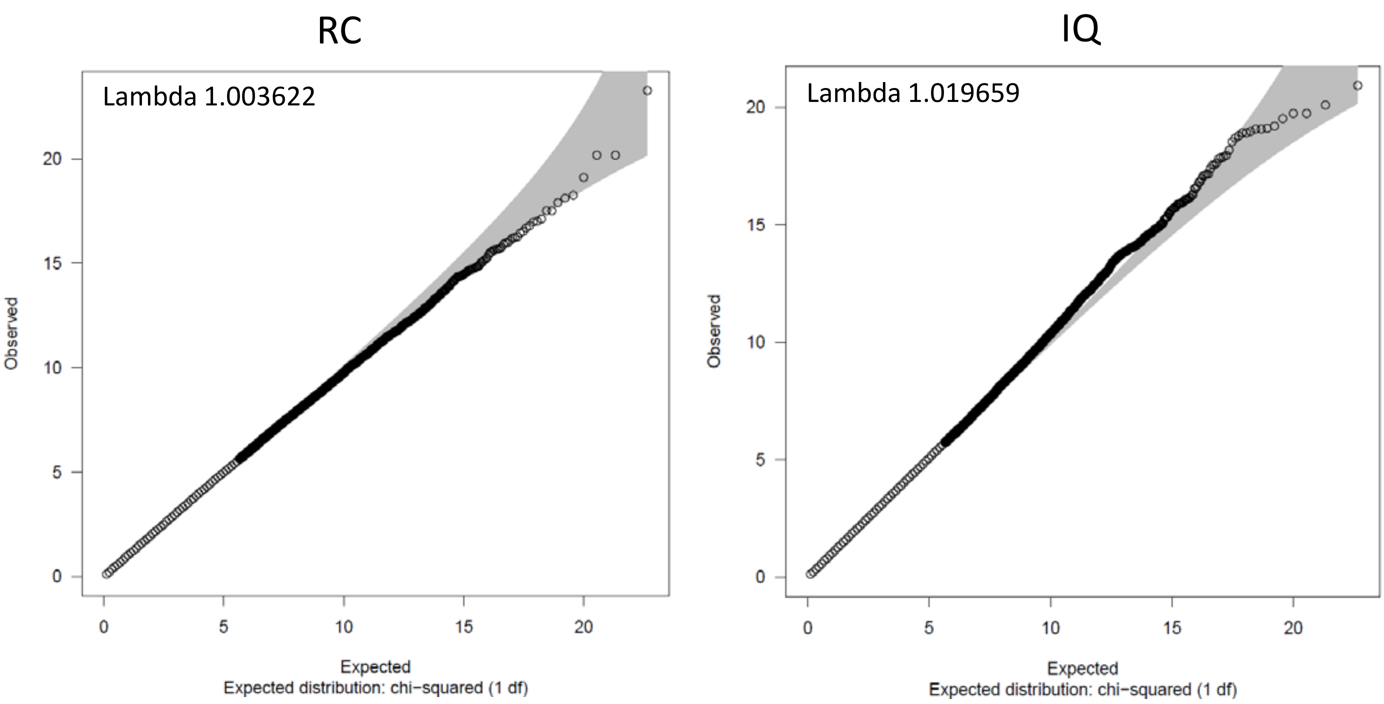

Figure S2. QQ plots for Relational Complexity (RC) and IQ show no evidence of population stratification, withLambda values indicating no inflation of association signal.
