# Supplementary material for: Genetic Basis of a Cognitive Complexity Metric
Source: PLoS One. 2015 Apr 10;10(4):e0123886. doi: 10.1371/journal.pone.0123886 (PMC4393228; doi:10.1371/journal.pone.0123886)
Supplement: S1 Table — (PDF) [file pone.0123886.s004.pdf]

Table S1. Methods: Discovery Sample Genotyping and Preliminary Analyses

Zygosity and Genotyping	Zygosity was determined from DNA using a commercial kit (AmpFlSTR Profiler Plus Amplification Kit, ABI) and was later confirmed in those genotyped. DNA samples were genotyped using the Illumina 610-Quadv1 whole-genome SNP array (San Diego, California) as described in detail elsewhere (20). Briefly, SNPs (total of 516,133) were filtered to have a mean GenCall score ≥ 0.7, call rate ≥ 95%, a minor allele frequency (MAF) ≥ 1%, and Hardy-Weinberg Equilibrium (HWE) ≥ 10^{-6}.
Data Transformation (in SPSS)	RC, N-term, latin square, and sentence comprehension were transformed to reduce negative skew using IBM SPSS Statistics Version 19. They were log transformed (log_{10}(K-X), where K was a constant from which each score was subtracted so that the smallest score was 1 and X was the score), with the exception of Sentence Comprehension, which was square root transformed (sqrt(K-X)). IQ, working memory, and reasoning were normally distributed. Distributions for the RC component before and after transformation: 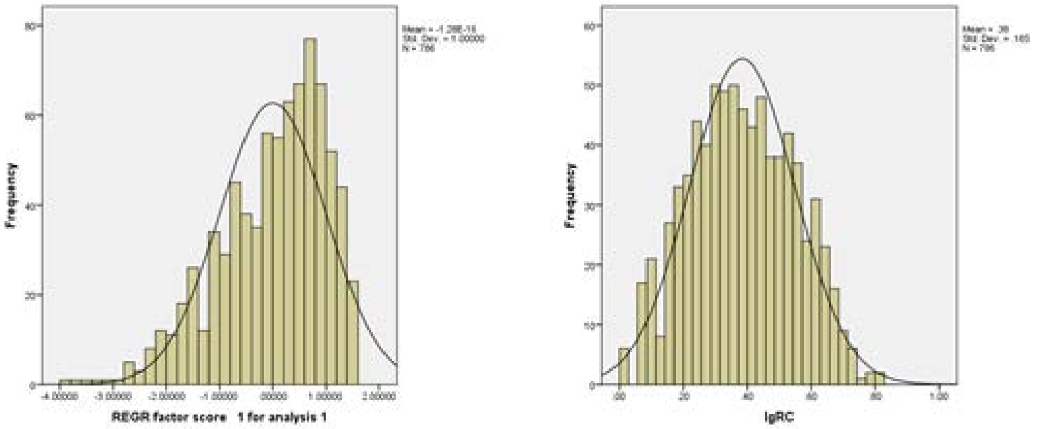
Modelling of Data (in Mx)	Modelling of raw data was performed in Mx using a full information maximum likelihood (FIML) estimator, which uses all data points regardless of missingness [1]. The fit of constrained models was compared to the full model by examining the difference in the -2 log likelihood, which is distributed as a chi-square for given degrees of freedom.
Assumption Testing (in Mx)	We assessed homogeneity of sampling by examining the means and variances for birth order and zygosity effects as described in McGregor et al. [2]. Note that for those twins who were not genome-scanned, zygosity was determined using a commercial kit (AmpFISTR Profiler Plus Amplification Kit, ABI).
Sex and Age Effects (in Mx)	The effects of sex and age were assessed by comparing the fit of models that either included, or excluded, them as covariates. Significant covariates were included in further modelling.
Test-retest Reliability (in Mx)	To examine test-retest reliability, data were collapsed over birth order and zygosity.
Twin Correlations (in Mx)	We tested if twin correlations for males and females could be set equal for (1) MZ and (2) DZ pairs. If not, this is suggestive of *magnitude* differences in genetic and/or environmental estimates for boys and girls. Similarly, if correlations for opposite-sex DZ pairs are significantly lower than those of same-sex DZ pairs, this indicates different *sources* of influence between boys and girls. Where DZ (and MZ) correlations could be set equal for males and females, and further, where opposite-sex DZ correlations could be set equal to same-sex DZ correlations, then further analyses were run with one MZ and one DZ group.
Significance levels (GWA and VEGAS) adjusted for correlated traits.	Significance levels for genome-wide association (GWA) and gene-based analyses (VEGAS) were adjusted for testing two correlated traits as per matSpD (http://gump.qimr.edu.au/general/daleN/matSpD). For GWA, the corrected threshold was $3.1x10^{-8}$ (standard threshold = $5.0x10^{-8}$). For VEGAS, the threshold after correcting for 17,668 genes (0.05/17,688) and two correlated traits was $1.7x10^{-6}$.

NOTE: RC = Relational Complexity, MZ = monozygotic, DZ = dizygotic

References

1. Neale MC, Cardon LR (1992) Methodology for genetic studies of twins and families. Dordrecht: Kluwer Academic Publishers.

2. McGregor B, Pfitzner J, Zhu G, Grace M, Eldridge A, et al. (1999) Genetic and environmental contributions to size, color, shape, and other characteristics of melanocytic naevi in a sample of adolescent twins. Genetic Epidemiology 16: 40-53.
