# Supplementary material for: Genetic Basis of a Cognitive Complexity Metric
Source: PLoS One. 2015 Apr 10;10(4):e0123886. doi: 10.1371/journal.pone.0123886 (PMC4393228; doi:10.1371/journal.pone.0123886)
Supplement: S2 Table — (PDF) [file pone.0123886.s005.pdf]

Table S2. Results for Assumption Testing and Sex and Age Effects.

**Analyses**	**Details**
Assumption Testing	No birth order or zygosity effects were found for any measure for means or variances ($\Delta\chi^2_4$ ranged 0.4-4.1 for birth order, $\Delta\chi^2_2$ ranged 0.04-1.5 for zygosity), with the exception of a variance difference for zygosity found for Reasoning ($\Delta\chi^2_2 = 8.6$), where female DZ pairs had low variability (0.66-0.69) compared to male and female MZ pairs and male DZ pairs (0.91-1.00). As a consistent zygosity effect across both males and females was not found, variances for female MZ and DZ pairs were set to be equal. Means and variances for non-twin siblings could be set equal to those of twins for all measures except the Sentence and N-term Tasks, where for males only, non-twin siblings had higher means than twins (18.2 vs 16.8, $\Delta\chi^2_2 = 8.1$ and 13.3 vs 12.0, $\Delta\chi^2_2 = 10.4$ respectively). Means for these measures were left free to vary between non-twin siblings and twins in further analyses. The differences found may reflect a sampling issue due to the small number of males in the non-twin sibling group (33 males vs. 67 females - note that 3rd born DZ triplets are grouped with non-twin siblings in analyses, as the genetic relationship is the same).
Sex and Age Effects	Means and variances could be set equal for males and females for all measures except IQ and Reasoning, where means differed significantly ($\Delta\chi^2_1 = 22.2$ and 12.6 respectively). On average, males had higher IQ scores than females (*M* = 113.9 vs. 108.7) and higher Reasoning scores (factor z-score *M* = 0.14 vs. -0.11). Consequently, sex was included as a covariate in further analyses. No effects for age were found ($\Delta\chi^2_1$ ranged 0.1-2.4).
