# Supplementary material for: Genetic Basis of a Cognitive Complexity Metric
Source: PLoS One. 2015 Apr 10;10(4):e0123886. doi: 10.1371/journal.pone.0123886 (PMC4393228; doi:10.1371/journal.pone.0123886)
Supplement: S3 Table — (PDF) [file pone.0123886.s006.pdf]

Table S3. Sample and Analyses Details for the Replication Cohorts

**English (Avon Longitudinal Study of Parents and Children (ALSPAC))**	
Sample	**This Study:** 4078 unrelated adolescents (47.4% male) aged 14-17 years (*M* = 15.4±0.3).<br>**Full Study Numbers:** ALSPAC recruited 14,541 pregnant women resident in Avon, UK with expected dates of delivery 1st April 1991 to 31st December 1992. 14,541 is the *initial* number of pregnancies for which the mother enrolled in the ALSPAC study and had either returned at least one questionnaire or attended a "Children in Focus" clinic by 19/07/99. Of these *initial* pregnancies, there was a total of 14,676 fetuses, resulting in 14,062 live births and 13,988 children who were alive at 1 year of age. When the oldest children were approximately 7 years of age, an attempt was made to bolster the initial sample with eligible cases who had failed to join the study originally. As a result, when considering variables collected from the age of seven onwards (and potentially abstracted from obstetric notes) there are data available for more than the 14,541 pregnancies mentioned above. The number of **new pregnancies** not in the initial sample (known as Phase I enrolment) that are currently represented on the built files and reflecting enrolment status at the age of 18 is 706 (452 and 254 recruited during Phases II and III respectively), resulting in an additional 713 children being enrolled. The phases of enrolment are described in more detail in the cohort profile papers:<br>1) Boyd A, Golding J, Macleod J, Lawlor DA, Fraser A, Henderson J, Molloy L, Ness A, Ring S, Davey Smith G. Cohort Profile: The 'Children of the 90s'--the index offspring of the Avon Longitudinal Study of Parents and Children Int J Epidemiol. 2013 Feb;42(1):111-27. PMID: 22507743 <http://ije.oxfordjournals.org/content/early/2012/04/14/ije.dys064.full.pdf+html>.<br>2) Fraser A, Macdonald-Wallis C, Tilling K, Boyd A, Golding J, Davey Smith G, Henderson J, Macleod J, Molloy L, Ness A, Ring S, Nelson SM, Lawlor DA. Cohort Profile: The Avon Longitudinal Study of Parents and Children: ALSPAC mothers cohort. Int J Epidemiol. 2013;42(1)97-110. PMID: 22507742 http://ije.oxfordjournals.org/content/42/1/97.full.pdf+html<br>The total sample size for analyses using any data collected after the age of seven is therefore 15,247 pregnancies, resulting in 15,458 fetuses. Of this **total sample** of 15,458 fetuses, 14,775 were **live births** and 14,701 were **alive at 1 year of age**. A 10% sample of the ALSPAC cohort, known as the **Children in Focus (CiF) group**, attended clinics at the University of Bristol at various time intervals between 4 to 61 months of age. The CiF group were chosen at random from the last 6 months of ALSPAC births (1432 families attended at least one clinic). Excluded were those mothers who had moved out of the area or were lost to follow-up, and those partaking in another study of infant development in Avon.
Genotyping (of the children)	A total of 9912 subjects were genotyped using the Illumina HumanHap550 quad genome-wide SNP genotyping platform by 23andMe subcontracting the Wellcome Trust Sanger Institute, Cambridge, UK and the Laboratory Corporation of America, Burlington, NC, USA. Individuals were excluded from further analysis on the basis of having incorrect gender assignments; minimal or excessive heterozygosity (<0.320 and >0.345 for the Sanger data and <0.310 and >0.330 for the LabCorp data); disproportionate levels of individual missingness (>3%); evidence of cryptic relatedness (>10% IBD) and being of non-European ancestry (as detected by a multidimensional scaling analysis seeded with HapMap 2 individuals, EIGENSTRAT analysis revealed no additional obvious population stratification and genome-wide analyses with other phenotypes indicate a low lambda). The resulting data set consisted of 8365 individuals. SNPs with a minor allele frequency of <1% and call rate of <95% were removed. Furthermore, only SNPs which passed an exact test of Hardy–Weinberg equilibrium (P>5×10-7) were considered for analysis. After quality control, 8365 unrelated individuals, who were genotyped at 500527 SNPs, were available for analysis. EIGENSTRAT principal components analysis was used to generate the top 100 principal components after the removal of known regions of long linkage disequilibrium in the data [1,2]. Known autosomal variants were imputed with MACH 1.0.16 Markov Chain Haplotyping software [3,4], using CEPH individuals from phase 2 of the HapMap project (HG18) as a reference set (release 22). For the X chromosomal variants, imputation was performed using MiniMac (v 4.43) [5] and CEPH individuals from phase 3 of the HapMap project (HG18) were used as the reference set.
Ethical Approval	Ethical approval for the study was obtained from the ALSPAC Ethics and Law Committee and the Local Research Ethics Committees.
Current Cohort	The cohort examined in the current study participated in the Teen Focus 3 (TF3) clinic conducted from October 2006 to November 2008, when they were aged from 14.3 to 17.7 years (*M*=15.5). There were 5515 participants in TF3, of whom 4078 had both genotyping and the phenotypes of interest.
Phenotypes Examined	Matrix Reasoning: a subtest of the WASI [6], and IQ: derived from the WASI subtests (Vocabulary, Similarities, Block Design, and Matrix Reasoning - note that IQ and Matrix Reasoning are not independent. Please note that the study website contains details of all the data that is available through a fully searchable data dictionary (http://www.bris.ac.uk/alspac/researchers/data-access/data-dictionary/).
Analyses	Sex, age, and 10 ancestry principal components were included as covariates in all analyses. SNP-based analyses of association were performed in PLINK [7] for 11 target SNPs as well as SNPs located in the genes *NPS* (55 SNPs) and *FAM105A* (30 SNPs). P-values obtained for the SNPs in the two genes were used to run gene-based analyses with the software VEGAS [Versatile Gene-Based Association Study; 8] to derive gene-based p-values.
**Scottish Cohort (Lothian Birth Cohort 1936 (LBC1936))**	
Sample	This sample is the University of Edinburgh-based Lothian Birth Cohort of 1936 [9]. Most of the participants undertook a general intelligence test (Moray House Test No. 12) at age 11 years and were recruited for this

	cohort at a mean age of 70. Assessments have been in three waves (Wave 1: mean age = 70 years; Wave 2: mean age =73; Wave 3: mean age = 76). In total, 1091 participants joined the LBC1936 and were assessed at Wave 1 (548 men, 543 women) for cognitive ability and other attributes. Both the phenotypes of interest and genotypes were available for 986-1001 individuals (varying with phenotype).
Phenotype Data	The phenotypes of interest were reasoning (Matrix Reasoning from the Wechsler Adult Intelligence Scale-III UK), working memory (Letter Number Sequencing from the WAIS-III UK), and general cognitive ability, or IQ (Moray House Test).
Genotyping	DNA was extracted from blood samples and SNP genotyping was performed with the Illumina 610k Quad Bead chip either by the Wellcome Trust Clinical Research Facility in Edinburgh. Individuals were checked for disagreement between genetic and reported gender. Relatedness between subjects was investigated and, for any related pair of individuals, one was removed. Samples with a call rate ≤.95, and those showing evidence of non-caucasian descent, were also removed. SNPs were included in the analyses if they met the following conditions: call rate ≥.98, minor allele frequency ≥.01, and HWE test with P ≥.001.
Analyses	The single SNP analysis of 11 target SNPs and SNPs located in the genes *NPS* and *FAM105A* was performed in PLINK [7] using a linear regression additive model. All cognitive phenotypes were corrected for age, sex and four MDS components prior to the analysis. Seven subjects with outlying Moray House Test scores (Z< -3.5) were removed prior to the Moray House Test analyses. There were no Matrix Reasoning or Letter Number Sequencing outliers. The gene based analysis was performed in VEGAS [8].
**Dutch Cohort (Netherlands Twin Registry (NTR))**	
Sample & Phenotype Data	The phenotype data were collected as part of several different projects within the NTR [10-12], and consisted of summed scores on the Raven's Standard Progressive Matrices or Raven's Advanced Progressive Matrices, for 920 individuals from 340 families. 44.7% of the sample was male. The sample contained mostly children (N=737, age: M=14.5, SD=3.2) and some adults (N=183, age: M=43.8, SD=3.9). Seeing as the data originated from different projects (with measurements taking place at children's age 9, 12, 15, or 17), the Raven scores were standardized within each project (M=0, SD=1). Part of the sample was measured longitudinally (on 2 measurement occasions, 3 years apart); for these individuals, the standardized data were averaged over measurement occasions. Standardized Raven scores were used in all subsequent analyses.
Genotype Data	Blood and/or buccal samples for DNA extraction were collected as part of several projects within the NTR. Genotyping was performed using the Affymetrix Human SNP Array 6.0 at the Avera Institute, Sioux Falls, South Dakota, USA. Genotypes were called using the BIRDSEED V2 algorithm. Samples were selected if the CQC metric was above .4 (resulting in a call rate > 99%) and checked for Mendelian errors, excessive heterozygosity, ethnic outliers and discrepancies in relatedness and gender. For the present study, we selected 10 SNPs (rs4390263, rs12882037, rs3827183, rs11195283, rs2442756, rs1242923, rs4482248, rs12419146, rs7801010, and rs2964546 (genotyping was not available for rs10209999), and all available SNPs from 2 genes (*NPS* and *FAM105A*; 32 and 41 SNPs, respectively).
Analyses	All analyses were performed using sex, age, information on type of sample used for DNA extraction (blood/buccal), and 12 principal components (10 representing ancestry and 2 representing chip effects) as covariates. The SNP-based analyses were performed in Plink [7], using the --family option to control for family structure. For the SNPs residing on the two genes of interest, the SNP-based p-values obtained using Plink were subsequently used as input for VEGAS [8] to obtain gene-based p-values.
**Norwegian Cohort (Norwegian Cognitive NeuroGenetics (NCNG))**	
Sample	This sample was recruited through newspaper advertisements in the Oslo and Bergen urban areas and comprised 670 unrelated individuals aged 20-79 years (*M*=47.6±18.3) with mean IQ 119±10.6 [13]. All participants were interviewed and probed for past or present neurological or psychiatric diseases known to affect the central nervous system, and for history of substance abuse. Any person with a history of treatment for any of these conditions was excluded from the sample. Participants should have completed basic education with no history of learning deficits; persons who, after initial inclusion, on subsequent testing scored more than one standard deviation (*SD*) below their age norm on intelligence or memory were excluded. Furthermore, persons with a score on a depression inventory indicating a previously undiagnosed depressive illness were excluded. The participants were native speakers of Norwegian. All participants gave their informed consent for participation, which included donation of a blood sample, DNA extraction and genotyping, and storage of the remaining blood sample in a biobank. The recruitment procedure resulted in a cognitively normal sample, skewed towards the high functioning intelligence range.
Genotyping	DNA samples were freshly extracted from blood and genotyped on the Illumina Human610-Quad Beadchip. Quality control was performed with the iterative 'check.marker' function in the R package GenABEL [14]. Cryptic relatedness was assessed by identity-by-state (ibs), removing one sample from a pair with 'ibs.threshold' ≥ .85. Individualswith heterozygosity values greater than two *SD*s from the sample mean, or with unresolved sex discrepancies, were removed. Population structure was assessed by multidimensional scaling (MDS) analysis (100K random single nucleotide polymorphisms (SNPs), removing outlying samples with possible recent non-Norwegian ancestry. Finally, SNPs with a call rate < .95, minor allele frequency (MAF) < .01, and Hardy-Weinberg Equilibrium (HWE) exact test P < .001, were excluded. This resulted in a final dataset of 554,225 SNPs genotyped in a homogeneous Norwegian sample of 670 individuals [13].
Measures	Data collected from a broad battery of psychometric tests provided measures including IQ (obtained from Vocabulary and Matrix Reasoning subtests from the Wechsler Abbreviated Scale of Intelligence [WASI; 6]), reasoning (WASI Matrix Reasoning), working memory (Letter-Number Span, Digit Symbol), and processing speed (Digit Symbol) [13].

	Note that the reasoning measure is not independent of the score for IQ.
Analyses	Association analyses were performed using PLINK [7] for 11 target SNPs and SNPs located in the genes *NPS* and *FAM105A*. Sex and age were included as covariates for all traits with the exception of IQ, for which sex was the only covariate. Gene-based analyses were performed using VEGAS [8].

References

1. Price AL, Patterson NJ, Plenge RM, Weinblatt ME, Shadick NA, et al. (2006) Principal components analysis corrects for stratification in genome-wide association studies. Nat Genet 38: 904-909.
2. Price AL, Weale ME, Patterson N, Myers SR, Need AC, et al. (2008) Long-range LD can confound genome scans in admixed populations. Am J Hum Genet 83: 132-135; author reply 135-139.
3. Li Y, Willer C, Sanna S, Abecasis G (2009) Genotype imputation. Annu Rev Genomics Hum Genet 10: 387-406.
4. Li Y, Willer CJ, Ding J, Scheet P, Abecasis GR (2010) MaCH: using sequence and genotype data to estimate haplotypes and unobserved genotypes. Genet Epidemiol 34: 816-834.
5. Howie B, Fuchsberger C, Stephens M, Marchini J, Abecasis GR (2012) Fast and accurate genotype imputation in genome-wide association studies through pre-phasing. Nat Genet 44: 955-959.
6. Wechsler D (1999) Wechsler Abbreviated Scale of Intelligence. San Antonio, TX: The Psychological Corporation.
7. Purcell S, Neale B, Todd-Brown K, Thomas L, Ferreira MA, et al. (2007) PLINK: a tool set for whole-genome association and population-based linkage analyses. Am J Hum Genet 81: 559-575.
8. Liu JZ, McRae AF, Nyholt DR, Medland SE, Wray NR, et al. (2010) A versatile gene-based test for genome-wide association studies. Am J Hum Genet 87: 139-145.
9. Deary IJ, Gow AJ, Pattie A, Starr JM (2012) Cohort profile: the lothian birth cohorts of 1921 and 1936. Int J Epidemiol 41: 1576-1584.
10. Groot AS, de Sonneville LM, Stins JF, Boomsma DI (2004) Familial influences on sustained attention and inhibition in preschoolers. J Child Psychol Psychiatry 45: 306-314.
11. Rijsdijk FV, Vernon PA, Boomsma DI (2002) Application of hierarchical genetic models to Raven and WAIS subtests: a Dutch twin study. Behav Genet 32: 199-210.
12. Van Leeuwen M, Van den Berg S, Boomsma DI (2008) A twin-family of general IQ. Learning and Individual Differences 18: 76-88.
13. Espeseth T, Christoforou A, Lundervold AJ, Steen VM, Le Hellard S, et al. (2012) Imaging and Cognitive Genetics: The Norwegian Cognitive NeuroGenetics Sample. Twin Research and Human Genetics 15: 442-452.
14. Aulchenko YS, Ripke S, Isaacs A, van Duijn CM (2007) GenABEL: an R library for genome-wide association analysis. Bioinformatics 23: 1294-1296.
