# Supplementary material for: Genetic Basis of a Cognitive Complexity Metric
Source: PLoS One. 2015 Apr 10;10(4):e0123886. doi: 10.1371/journal.pone.0123886 (PMC4393228; doi:10.1371/journal.pone.0123886)
Supplement: S4 Table — (PDF) [file pone.0123886.s007.pdf]

Table S4. Univariate Cholesky Decomposition showing Additive Genetic (A), Common Environmental (C), and Non-shared Environmental Influences (E) (with 95% Confidence Intervals).

Model	-2LL	df	Δ-2LL	Δdf	AIC	A	C	E
Latin Square								
ACE	2188.9	781	-	-	626.9	0.44 (0.16, 0.55)	0.00 (0.00, 0.20)	0.56 (0.45, 0.69)
**AE**	**2188.9**	**782**	**0.0**	**1**	**624.9**	**0.44 (0.31, 0.55)**	-	**0.56 (0.45, 0.69)**
CE	2196.9	782	8.0	1	632.9	-	0.28 (0.18, 0.37)	0.72 (0.63, 0.82)
E	2230.5	783	41.6	2	664.5	-	-	1.00 (1.00, 1.00)
N-term								
ACE	2157.6	779	-	-	599.6	0.31 (0.00, 0.57)	0.16 (0.00, 0.40)	0.53 (0.42, 0.67)
**AE**	**2159.3**	**780**	**1.7**	**1**	**599.3**	**0.50 (0.39, 0.60)**	**-**	**0.50 (0.40, 0.61)**
CE	2161.0	780	3.4	1	601.0	-	0.37 (0.28, 0.45)	0.63 (0.55, 0.72)
E	2219.3	781	61.6	2	657.3	-	-	1.00 (1.00, 1.00)
Sentence								
ACE	2152.6	780	-	-	592.6	0.48 (0.18, 0.64)	0.06 (0.00, 0.29)	0.45, 0.36, 0.58)
**AE**	**2152.9**	**781**	**0.3**	**1**	**590.9**	**0.56 (0.45, 0.65)**	-	**0.44 (0.35, 0.55)**
CE	2161.8	781	9.2	1	599.8	-	0.38 (0.29, 0.46)	0.62 (0.54, 0.71)
E	2224.0	782	71.4	2	660.0	-	-	1.00 (1.00, 1.00)
Relational Complexity								
ACE	2110.2	779	-	-	552.2	0.61 (0.36, 0.75)	0.07 (0.00, 0.27)	0.32 (0.25, 0.42)
**AE**	**2110.6**	**780**	**0.4**	**1**	**550.6**	**0.68 (0.60, 0.75)**	**-**	**0.32 (0.25, 0.40)**
CE	2129.6	780	19.5	1	569.6	-	0.46 (0.38, 0.54)	0.54 (0.46, 0.62)
E	2224.5	782	114.3	2	662.5	-	-	1.00 (1.00, 1.00)
IQ								
ACE	1935.9	774	-	-	387.9	0.72 (0.54, 0.87)	0.12 (0.00, 0.30)	0.16 (0.12, 0.20)
**AE**	**1937.4**	**775**	**1.5**	**1**	**387.4**	**0.84 (0.80, 0.88)**	**-**	**0.16 (0.12, 0.20)**
CE	1993.1	775	57.2	1	443.1	-	0.62 (0.55, 0.68)	0.38 (0.32, 0.45)
E	2180.2	776	244.3	2	628.2	-	-	1.00 (1.00, 1.00)
Working Memory								
**ACE**	2035.5	752	-	-	531.5	0.52 (0.25, 0.71)	0.11 (0.00, 0.32)	0.37 (0.29, 0.47)
**AE**	**2036.5**	**753**	**1.0**	**1**	**530.5**	**0.64 (0.55, 0.72)**	-	**0.36 (0.28, 0.45)**
CE	2049.6	753	14.1	1	543.6	-	0.46 (0.38, 0.54)	0.54 (0.46, 0.62)
E	2143.0	754	107.4	2	635.0	-	-	1.00 (1.00, 1.00)
Reasoning								
ACE	2008.2	749	-	-	510.2	0.46 (0.20, 0.68)	0.16 (0.00, 0.37)	0.38 (0.30, 0.48)
**AE**	**2010.3**	**750**	**2.0**	**1**	**510.3**	**0.63 (0.55, 0.70)**	**-**	**0.37 (0.30, 0.45)**
CE	2020.5	750	12.2	1	520.5	-	0.48 (0.40, 0.56)	0.52 (0.45, 0.60)
E	2130.7	751	122.5	2	628.7	-	-	1.00 (1.00, 1.00)

NOTE: RC = Relational Complexity, IQ =Full-scale IQ, WMem = Working Memory
[a]Best-fitting models shown in **bold**.
