# Supplementary material for: Genetic Basis of a Cognitive Complexity Metric
Source: PLoS One. 2015 Apr 10;10(4):e0123886. doi: 10.1371/journal.pone.0123886 (PMC4393228; doi:10.1371/journal.pone.0123886)
Supplement: S5 Table — (PDF) [file pone.0123886.s008.pdf]

Table S5. Multivariate Model-Fitting Results (best-fitting model in **bold,** parameter estimates shown for best-fitting model) for Latin Square, N-term, and Sentence Tasks.

Model[a]	-2 log likelihood	df	AIC
1. Cholesky ACE	6051.391	2331	1389.4
**2. Cholesky AE**	**6052.943**	**2337**	**1378.9**
3. Cholesky CE	6075.994	2337	1402.0
4. Independent Pathway: 1xA Factor plus Specifics, Cholesky E	6056.261	2237	1382.3
5. Common Pathway: 1 Common Factor, plus specifics	6057.401	2339	1379.4

	Cholesky AE Model Parameters (shown as a %)							
	Additive Genetic Factors[b]			Total A	Unshared Environmental Factors[b]			
	A1	A2	A3	(h^2)	E1	E2	E3	Total E
Sentence	55 (45-64)	-	-	55	45 (36-55)	-	-	45
N-term	40 (29-51)	11 (03-21)	-	51	02 (0.1-05)	47 (39-56)	-	49
Latin Square	18 (05-29)	23 (07-36)	01 (00-18)	43	01 (00-04)	00 (00-0.1)	56 (46-67)	57

[a]The significance of additive genetic (A) and common environmental (C) influences were tested in a Cholesky model. While C could be dropped from the fully-saturated model without loss of fit ($\Delta\chi^2_6$ = 1.6; Model 2), dropping A resulted in a significant worsening of fit ($\Delta\chi^2_6$ = 24.6; Model 3). Therefore, a Cholesky model allowing for A and E, but not C influences (i.e. Model 2) was the most parsimonious and best-fitting Cholesky model. Retaining the AE format, independent and common pathway models were compared to the best-fitting Cholesky model. The AE Cholesky remained the best-fitting, although the fit of the common pathway model was almost identical (AIC = 1378.9 vs. 1379.4), and consequently, estimates are shown for both models (Cholesky in this Table and the common pathway, which is considered a more interpretable model [1], in Figure 2.
[b]Cholesky estimates on the diagonal include both common and specific influences (A1, A2, E1, E2) or specific influences only (A3, E3). Estimates on the off-diagonal represent common influences

References

1. Loehlin JC (1996) The Cholesky approach: A cautionary note. Behavior Genetics 26: 65-69.
