# Supplementary material for: Genetic Basis of a Cognitive Complexity Metric
Source: PLoS One. 2015 Apr 10;10(4):e0123886. doi: 10.1371/journal.pone.0123886 (PMC4393228; doi:10.1371/journal.pone.0123886)
Supplement: S6 Table — (PDF) [file pone.0123886.s009.pdf]

Table S6. Top 50 Single Nucleotide Polymorphisms (SNPs)[a] for Relational Complexity (RC)

Chr	SNP	AL1	AL2	MAF (AL1)	Beta	SE	*p*-value	Gene	*p*-values in Related Traits		
									IQ	Reasoning	Working Memory
**10**	**rs4390263**	A	G	.449	-.348	.072	1.4×10^{-6}	near NPS[c]	2.4×10^{-2}	3.3×10^{-3}	4.5×10^{-2}
11	rs12807847	T	C	.058	.667	.152	1.2×10^{-5}	SLC5A12	6.4×10^{-3}	.080	.059
3	rs7625359	T	C	.106	-.496	.116	1.9×10^{-5}	-	.961	.133	.192
9	rs10820743	C	T	.303	-.302	.071	2.1×10^{-5}	ABCA1	.207	.152	.012
11	rs4755955	A	G	.176	-.415	.098	2.3×10^{-5}	-	3.1×10^{-2}	.147	.091
17	rs3606	A	G	.344	.319	.077	3.5×10^{-5}	SLC39A11	.488	4.9×10^{-2}	1.4×10^{-2}
9	rs722353	C	T	.175	.376	.091	3.7×10^{-5}	ZNF169	.098	2.9×10^{-2}	.320
**14**	**rs12882037**	T	C	.232	.356	.086	3.7×10^{-5}	-	3.7×10^{-2}	5.6×10^{-3}	2.2×10^{-2}
14	rs4982279	G	T	.209	.353	.086	4.4×10^{-5}	-	1.3×10^{-2}	21.2×10^{-3}	.123
13	rs9567377	T	G	.117	-.436	.107	4.8×10^{-5}	-	.401	8.2×10^{-3}	1.0×10^{-3}
2	rs1905925	T	C	.485	.287	.071	5.5×10^{-5}	LRP1B	.263	1.3×10^{-3}	.064
20	rs2208981	C	T	.188	.346	.086	5.6×10^{-5}	-	.603	1.6×10^{-3}	4.4×10^{-2}
15	rs1565866	T	G	.440	.283	.070	5.7×10^{-5}	BUB1B	.063	5.8×10^{-2}	.134
15	rs1968813	A	C	.486	-.279	.070	6.4×10^{-5}	-	.091	1.1×10^{-3}	.094
2	rs17842284	A	G	.204	.343	.086	7.2×10^{-5}	-	.057	.224	.024
12	rs10777776	C	A	.374	.301	.076	7.4×10^{-5}	ELK3	.123	.161	3.2×10^{-2}
5	rs347721	T	C	.495	.279	.070	7.5×10^{-5}	CDH18	.798	3.4×10^{-2}	1.1×10^{-2}
9	rs12346832	C	T	.265	.318	.080	7.7×10^{-5}	-	.359	4.8×10^{-2}	4.6×10^{-2}
8	rs1499370	G	A	.381	.281	.071	7.7×10^{-5}	PVT1	.687	1.5×10^{-2}	.308
15	rs2175462	T	C	.290	-.317	.080	8.2×10^{-5}	-	.883	3.5×10^{-3}	1.9×10^{-3}
14	rs4561383	C	T	.167	.381	.097	8.6×10^{-5}	-	.060	.080	.220
11	rs12270727	T	G	.043	.699	.179	9.3×10^{-5}	SLC5A12	3.1×10^{-2}	.388	3.6×10^{-2}
17	rs7406894	T	C	.300	.295	.076	9.8×10^{-5}	-	.268	.332	.269
12	rs7315837	G	A	.204	-.353	.091	1.0×10^{-4}	-	.510	.107	8.9×10^{-4}
2	rs2724855	A	G	.381	.282	.073	1.0×10^{-4}	-	.057	5.5×10^{-3}	.420
17	rs4148418	G	A	.393	-.267	.069	1.1×10^{-4}	ANKRD40	.087	1.0×10^{-2}	.124
3	rs13079094	T	C	.324	-.289	.075	1.1×10^{-4}	-	.065	5.9×10^{-3}	1.1×10^{-2}
12	rs596940	C	T	.068	-.504	.131	1.2×10^{-4}	PITPNM2	3.5×10^{-2}	9.1×10^{-3}	.607
**21**	**rs3827183**	A	G	.128	-.406	.106	1.2×10^{-4}	DOPEY2	4.0×10^{-3}	3.1×10^{-5}	1.6×10^{-2}
17	rs757597	A	G	.440	.277	.072	1.2×10^{-4}	MYOCD	.437	2.8×10^{-2}	9.1×10^{-3}
4	rs1817186	A	G	.078	.569	.148	1.2×10^{-4}	-	.130	2.8×10^{-3}	3.1×10^{-2}
7	rs10243024	A	G	.248	-.328	.085	1.2×10^{-4}	MET	.137	1.3×10^{-2}	5.8×10^{-3}
16	rs1376047	T	C	.156	-.366	.095	1.2×10^{-4}	-	.167	.137	3.5×10^{-3}
1	rs4503375	G	A	.170	-.375	.098	1.3×10^{-4}	VAV3	.654	9.1×10^{-3}	1.9×10^{-2}
9	rs11791976	G	A	.058	-.605	.158	1.3×10^{-4}	-	.910	.142	.201
14	rs11624232	T	C	.164	.379	.099	1.3×10^{-4}	-	.157	2.3×10^{-2}	.058
21	rs2825236	C	T	.307	-.318	.083	1.4×10^{-4}	-	.608	.118	.874
9	rs273472	G	A	.432	-.272	.071	1.4×10^{-4}	-	.068	.126	1.2×10^{-2}
**2**	**rs10209999**	G	A	.229	-.303	.080	1.4×10^{-4}	-	3.3×10^{-2}	2.3×10^{-2}	8.0×10^{-3}
20	rs6109686	A	G	.151	.371	.098	1.4×10^{-4}	SPTLC3	4.1×10^{-2}	2.3×10^{-3}	.235
**10**	**rs11195283**	A	C	.293	-.281	.074	1.4×10^{-4}	RBM20	4.8×10^{-2}	1.4×10^{-2}	2.9×10^{-3}
**8**	**rs2442756**	C	A	.357	.282	.074	1.5×10^{-4}	VPS13B	2.5×10^{-4}	1.6×10^{-5}	4.1×10^{-2}
15	rs2665111	C	T	.296	.305	.080	1.5×10^{-4}	-	.342	4.1×10^{-3}	1.4×10^{-4}
17	rs792766	A	G	.400	.276	.073	1.5×10^{-4}	-	.066	.069	.661
17	rs1990293	A	G	.191	.344	.091	1.5×10^{-4}	BCAS3	.750	3.4×10^{-3}	.565
9	rs10821076	A	G	.479	.259	.068	1.5×10^{-4}	-	.084	1.4×10^{-2}	.054
2	rs1531078	T	G	.201	.357	.094	1.5×10^{-4}	-	.330	.081	.263
9	rs182719	A	G	.111	-.427	.113	1.6×10^{-4}	PTPRD	.190	4.6×10^{-2}	.160
2	rs6725328	G	A	.190	-.327	.087	1.6×10^{-4}	-	.311	2.3×10^{-5}	1.3×10^{-2}
13	rs1323103	G	A	.364	-.272	.072	1.7×10^{-4}	-	.823	8.9×10^{-3}	5.1×10^{-3}

[a]Retained if LD threshold < .5
[b]GWAS p-value < 0.05. Shown in **red** if at least nominally significant for all traits.
[c]Intronic SNP, downstream of NPS (dist=3.62kb), LD = .6
