# Supplementary material for: Genetic Basis of a Cognitive Complexity Metric
Source: PLoS One. 2015 Apr 10;10(4):e0123886. doi: 10.1371/journal.pone.0123886 (PMC4393228; doi:10.1371/journal.pone.0123886)
Supplement: S7 Table — (PDF) [file pone.0123886.s010.pdf]

Table S7. Top 50 Single Nucleotide Polymorphisms (SNPs)[a] for IQ

Chr	SNP	AL1	AL2	MAF (AL1)	Beta	SE	*p*-value	Gene	*p*-values in Related Traits		
									Relational Complexity	Reasoning	Working Memory
14	rs1242923	T	C	.388	-.165	.036	4.8×10^{-6}	ABHD4	.121	.711	.911
2	rs4438512	G	A	.442	-.153	.034	8.8×10^{-6}	ZNF638	.135	.187	.168
3	rs6777812	T	C	.239	-.179	.041	1.0×10^{-5}	COLQ	.091	.414	.511
10	rs2804164	T	C	.229	-.175	.040	1.2×10^{-5}	ATRNL1	.826	.786	.407
6	rs1570086	T	C	.426	-.152	.035	1.2×10^{-5}	-	.258	.173	.615
5	rs152642	G	T	.139	-.218	.050	1.3×10^{-5}	-	1.2×10^{-2}	6.3×10^{-3}	.293
7	rs4333513	T	C	.231	-.177	.041	1.4×10^{-5}	-	.398	.635	.694
4	rs1055263	A	G	.188	.193	.045	1.5×10^{-5}	-	3.7×10^{-3}	.062	.235
**15**	**rs4482248**	A	G	.223	-.177	.041	1.7×10^{-5}	-	1.8×10^{-3}	4.2×10^{-5}	3.3×10^{-3}
11	rs2851682	G	A	.087	.265	.062	2.0×10^{-5}	FADS2	.138	1.8×10^{-2}	.201
22	rs2023635	G	A	.308	-.158	.037	2.3×10^{-5}	-	.220	.139	.662
18	rs948699	A	C	.051	-.346	.082	2.3×10^{-5}	-	.298	.171	.137
6	rs4035344	G	A	.129	.213	.051	2.7×10^{-5}	C6orf211	.106	.185	.203
1	rs1726672	T	C	.289	.163	.039	2.8×10^{-5}	-	.725	.323	.416
**11**	**rs12419146**	A	C	.042	.370	.089	3.0×10^{-5}	PRR5L	6.2×10^{-4}	3.1×10^{-4}	2.5×10^{-3}
5	rs247456	C	T	.171	.187	.045	3.4×10^{-5}	-	4.4×10^{-2}	.129	.604
7	rs13242229	C	T	.307	-.158	.038	3.4×10^{-5}	DGKB	.126	7.8×10^{-3}	6.1×10^{-3}
21	rs3746882	C	T	.125	.221	.053	3.5×10^{-5}	ETS2	.342	.153	.768
16	rs7201962	G	A	.247	.164	.040	3.6×10^{-5}	-	.131	.541	.649
6	rs2496509	A	C	.350	.151	.037	3.8×10^{-5}	RIMS1	.772	.500	.832
5	rs7726354	T	C	.053	.329	.080	4.1×10^{-5}	-	.795	.733	.812
**7**	**rs7801010**	C	T	.276	.159	.039	4.5×10^{-5}	DGKB	5.3×10^{-3}	5.1×10^{-3}	1.7×10^{-2}
1	rs1436750	A	G	.071	-.269	.067	5.5×10^{-5}	CACHD1	.074	.054	.455
19	rs12460133	G	T	.261	.153	.038	5.6×10^{-5}	-	.343	.816	.814
20	rs804544	C	A	.067	.289	.072	5.8×10^{-5}	-	.125	1.9×10^{-2}	3.8×10^{-2}
5	rs9292673	G	A	.168	-.185	.046	5.8×10^{-5}	-	.410	3.8×10^{-2}	.051
7	rs4909242	G	A	.098	-.237	.059	6.1×10^{-5}	PTPRN2	.294	2.6×10^{-2}	.731
4	rs10031105	C	T	.200	-.173	.043	6.1×10^{-5}	-	.623	.723	.914
2	rs7592135	A	G	.369	.140	.035	6.1×10^{-5}	-	.662	.112	.222
2	rs10932241	C	A	.400	-.140	.035	6.4×10^{-5}	-	4.2×10^{-2}	.541	.615
6	rs12524770	T	C	.281	-.158	.039	6.5×10^{-5}	-	.067	3.4×10^{-3}	.395
5	rs17842609	A	C	.051	.310	.078	6.7×10^{-5}	-	.105	.196	.955
**5**	**rs2964546**	T	C	.332	.148	.037	6.7×10^{-5}	-	1.8×10^{-2}	4.4×10^{-2}	2.6×10^{-2}
14	rs11157695	G	A	.387	-.142	.036	6.7×10^{-5}	ABHD4	.346	.595	.278
6	rs311232	T	C	.176	-.181	.045	6.7×10^{-5}	BEND3	.101	1.9×10^{-2}	.105
14	rs17254544	A	G	.198	.176	.044	6.8×10^{-5}	-	.242	.164	.407
6	rs2791333	G	A	.384	.142	.036	7.2×10^{-5}	ZNF192	.460	.285	.846
7	rs17170988	C	A	.197	-.174	.044	7.4×10^{-5}	ELMO1	.886	.590	.677
5	rs17386472	T	C	.089	-.248	.063	7.5×10^{-5}	GDNF	.606	.997	.175
14	rs17128136	A	G	.075	-.265	.067	7.6×10^{-5}	SOCS4	.063	.050	.072
19	rs11672523	G	A	.115	-.210	.053	7.7×10^{-5}	SPTBN4	.057	1.7×10^{-3}	6.7×10^{-3}
1	rs12116428	T	C	.070	-.261	.066	7.9×10^{-5}	-	.616	.805	.382
18	rs9944757	T	G	.090	.241	.061	8.3×10^{-5}	-	.391	.614	.196
6	rs7765204	G	A	.318	-.145	.037	8.4×10^{-5}	-	.116	.609	.286
4	rs317892	A	G	.213	-.170	.043	8.9×10^{-5}	-	.113	.383	.551
1	rs963208	T	C	.352	-.141	.036	9.2×10^{-5}	-	.157	4.9×10^{-2}	.346
18	rs7233676	T	C	.363	.140	.036	9.2×10^{-5}	-	.655	.072	.766
3	rs2455826	T	G	.276	-.152	.039	9.3×10^{-5}	BTD	.815	.828	.548
1	rs3767004	A	G	.073	-.270	.069	9.6×10^{-5}	CACNA1E	.812	.843	.616
15	rs16967271	A	G	.055	.283	.073	1.0×10^{-4}	-	.174	.393	.499

[a]Retained if LD threshold < .5
[b]GWAS p-value < 0.05. Shown in **red** if at least nominally significant for all traits.
