# Supplementary material for: Genetic Basis of a Cognitive Complexity Metric
Source: PLoS One. 2015 Apr 10;10(4):e0123886. doi: 10.1371/journal.pone.0123886 (PMC4393228; doi:10.1371/journal.pone.0123886)
Supplement: S8 Table — (PDF) [file pone.0123886.s011.pdf]

Table S8. Minor Allele Frequencies for Follow-Up Single Nucleotide Polymorphisms (SNPs) in Discovery and Replication Samples

**Chr**	**SNP**	**Effect Allele**	**Non-Effect Allele**	**Minor Allele Frequency**				
				**Australian Discovery**	**Norwegian NCNG**	**Scottish LBC1936**	**Dutch NTR**	**English ALSPAC**
2	rs10209999	G	A	0.229	0.236	0.234	-	0.232
5	rs2964546	T	C	0.332	0.299	0.317	0.311	0.328
7	rs7801010	C	T	0.276	0.309	0.284	0.274	0.275
8	rs2442756	C	A	0.357	0.326	0.400	0.392	0.363
10	rs11195283	A	C	0.293	0.285	0.301	0.326	0.286
10	rs4390263	A	G	0.449	0.453	0.474	0.482	0.468
11	rs12419146	A	C	0.042	0.030	0.045	0.034	0.042
14	rs1242923	T	C	0.388	0.409	0.399	0.390	0.386
14	rs12882037	T	C	0.232	0.260	0.232	0.223	0.224
15	rs4482248	A	G	0.223	0.232	0.228	0.222	0.230
21	rs3827183	A	G	0.128	0.102	0.121	0.147	0.123
