# Supplementary material for: Genetic Basis of a Cognitive Complexity Metric
Source: PLoS One. 2015 Apr 10;10(4):e0123886. doi: 10.1371/journal.pone.0123886 (PMC4393228; doi:10.1371/journal.pone.0123886)
Supplement: S9 Table — (PDF) [file pone.0123886.s012.pdf]

Table S9. Association Results for Six Loci Selected for Replication from the Relational Complexity (RC) Genome-Wide Association Analysis

	rs10209999 (*intergenic*, Chr 2)			rs2442756 (*VPS13B*, Chr 8)			rs11195283 (*RBM20*, Chr 10)			rs4390263[a] (*near NPS*, Chr 10)			rs12882037 (*intergenic*, Chr 14)			rs3827183 (*DOPEY2*, Chr 21)		
	P value	β	SE	P value	β	SE	P value	β	SE	P value	β	SE	P value	β	SE	P value	β	SE
Discovery Sample (N ranges 481-1999 (234-894 families))																		
RC	***1.4x10^{-4}***	-0.30	0.08	***1.5x10^{-4}***	0.28	0.07	***1.4x10^{-4}***	-0.28	0.07	***1.4x10^{-6}***	-0.35	0.07	***3.7x10^{-5}***	0.36	0.09	***1.2x10^{-4}***	-0.41	0.11
IQ	***0.033***	-0.09	0.04	***2.5x10^{-4}***	0.13	0.04	***0.048***	-0.07	0.04	***0.024***	-0.08	0.04	***0.037***	0.09	0.04	***4.0x10^{-3}***	-0.15	0.05
Reasoning	***0.023***	-0.19	0.08	***1.6x10^{-5}***	0.33	0.08	***0.014***	-0.19	0.08	***3.3x10^{-3}***	-0.21	0.07	***5.6x10^{-3}***	0.24	0.09	***3.1x10^{-5}***	-0.44	0.11
Working Memory	***8.0x10^{-3}***	-0.21	0.08	***0.041***	0.15	0.07	***2.9x10^{-3}***	-0.22	0.07	***0.045***	-0.014	0.07	***0.022***	0.20	0.09	***0.016***	-0.25	0.10
English ALSPAC (N=4078 unrelated)																		
IQ	0.765	0.01	0.03	0.845	-0.01	0.02	0.534	0.02	0.02	0.856	-0.004	0.02	0.684	-0.01	0.03	0.754	-0.01	0.03
Matrix Reasoning	0.584	-0.01	0.03	0.354	0.02	0.02	0.888	-0.004	0.02	0.877	0.004	0.02	0.491	0.02	0.03	0.630	-0.02	0.03
Scottish LBC1936 ((N=1001 unrelated)																		
Moray House (IQ)	0.503	0.02	0.03	0.134	-0.05	0.03	0.203	-0.04	0.03	***0.041***	0.07	0.03	0.322	0.03	0.03	0.434	-0.02	0.03
Matrix Reasoning	0.884	-0.005	0.03	0.861	0.01	0.03	0.806	-0.01	0.03	***9.5x10^{-3}***	0.08	0.03	0.780	-0.01	0.03	0.750	0.01	0.03
Letter Number Sequence	0.847	0.01	0.03	0.268	-0.04	0.03	0.445	-0.02	0.03	**0.098**	0.05	0.03	0.663	0.01	0.03	0.716	-0.01	0.03
Dutch NTR (N=920 (340 families))																		
Raven's Prog. Matrices	-	-	-	0.162	-0.08	0.05	0.144	0.08	0.05	0.400	0.04	0.05	0.150	0.10	0.07	0.252	-0.09	0.08
Norwegian NCNG (N=670 unrelated)																		
IQ	0.210	-0.88	0.70	0.32	-0.63	0.63	0.129	0.99	0.65	**0.070**	1.08	0.59	**0.058**	-1.25	0.66	***0.029***	2.13	0.97
Matrix Reasoning	***0.045***	-0.52	0.26	***0.020***	-0.55	0.23	**0.096**	0.40	0.24	**0.081**	0.39	0.22	0.225	-0.30	0.24	0.128	0.55	0.36
Letter Number Span	0.106	-0.35	0.21	0.736	-0.06	0.19	0.157	0.28	0.20	0.512	-0.12	0.18	**0.095**	-0.33	0.20	***3.0x10^{-3}***	0.88	0.30
Digit Symbol	0.699	0.27	0.69	0.834	-0.13	0.62	0.475	-0.46	0.64	***2.0x10^{-3}***	1.81	0.58	0.268	0.72	0.65	0.310	-0.98	0.96

Combined Samples
Meta-analyses (IQ: N=7083 unrelated, Reasoning: N=6570 unrelated, Working Memory: N=1825 unrelated)

	rs10209999		rs2442756		rs11195283		rs4390263		rs12882037		rs3827183	
	P value	z-score	P value	z-score	P value	z-score	P value	z-score	P value	z-score	P value	z-score
IQ[b]	0.489	-0.693	0.755	0.312	0.791	-0.265	0.696	0.391	0.805	0.247	0.352	-0.931
Reasoning[c]	**0.052**	-1.943	0.637	0.472	0.943	-0.071	0.322	0.991	0.676	-0.419	0.417	-0.812
Working Memory[d]	0.558	-0.586	0.621	-0.495	0.496	-0.681	***0.023***	2.270	**0.076**	1.775	**0.088**	-1.710
	(**0.092**)	(-1.687)	(0.789)	(0.267)	(0.346)	(-0.943)	(0.385)	(0.869)	(0.762)	(0.303)	(0.690)	(0.399)

NOTE: P values <0.05 are shown in bold and underlined, while those >0.05 but <0.10 are shown in bold only. At a gene-based level, in the Discovery sample, *NPS* was the top ranked gene (p=1.5x10^{-5}) for RC, while *VPS13B* and *DOPEY2* were nominally associated (p=0.02, 0.04 respectively). *RBM20* was not a VEGAS-listed gene. Traits are standardised (z-scores, M=0±1) for all cohorts excepting NCNG.

[a] The top-ranked loci for RC was rs4390263.

[b] Meta-analysis for IQ included the following measures: Discovery - IQ (5 subtests of the Multidimensional Aptitude Battery), ALSPAC and NCNG - IQ (2 subtests of the WASI - includes Matrix Reasoning); LBC1936 - Moray House

[c] Meta-analysis for reasoning included the following measures: Discovery – RC; ALSPAC/LBC1936/NCNG - Matrix Reasoning; NTR - Raven's Progressive Matrices

[d] Meta-analysis for working memory included the following measures: Discovery – Working Memory component; LBC1936 – Letter Number Sequence; NCNG – Digit Symbol (results using Letter Number Span are shown in brackets)
