# Supplementary material for: Genetic Basis of a Cognitive Complexity Metric
Source: PLoS One. 2015 Apr 10;10(4):e0123886. doi: 10.1371/journal.pone.0123886 (PMC4393228; doi:10.1371/journal.pone.0123886)
Supplement: S10 Table — (PDF) [file pone.0123886.s013.pdf]

Table S10. Association Results for Four Loci Selected for Replication from the IQ Genome-Wide Association Analysis

	rs2964546 (*intergenic*, Chr 5)			rs7801010 (*DGKB*, Chr 7)			rs12419146 (*PRR5L*, Chr 11)			rs1242923[a] (*ABHD4*, Chr 14)			rs4482248 (*intergenic*, Chr 15)		
	**P value**	**β**	**SE**	**P value**	**β**	**SE**	**P value**	**β**	**SE**	**P value**	**β**	**SE**	**P value**	**β**	**SE**
Discovery Sample (N ranges 481-1999 (234-894 families))															
IQ	***$6.7x10^{-5}$***	0.15	0.04	***$4.5x10^{-5}$***	0.16	0.08	***$3.0x10^{-5}$***	0.37	0.09	***$4.8x10^{-6}$***	-0.17	0.04	***$1.7x10^{-5}$***	-0.18	0.04
Relational Complexity	***0.018***	0.17	0.07	***$5.3x10^{-3}$***	0.22	0.08	***$6.2x10^{-4}$***	0.57	0.17	0.121	0.11	0.07	***$1.8x10^{-3}$***	-0.26	0.08
Reasoning	***0.044***	0.15	0.07	***$5.1x10^{-3}$***	0.22	0.08	***$5.3x10^{-4}$***	0.62	0.17	0.711	-0.17	0.07	***$4.2x10^{-5}$***	-0.35	0.09
Working Memory	***0.026***	0.16	0.07	***0.017***	0.18	0.08	***$2.5x10^{-3}$***	0.51	0.17	0.911	0.01	0.07	***$3.3x10^{-3}$***	-0.24	0.08
English ALSPAC (N=4078 unrelated)															
IQ	0.139	0.04	0.02	0.941	-0.002	0.03	0.993	-0.001	0.06	0.489	-0.02	0.02	***0.021***	-0.06	0.03
Matrix Reasoning	0.112	-0.04	0.02	0.636	-0.01	0.03	0.881	-0.01	0.06	0.814	-0.005	0.02	**0.062**	0.05	0.03
Scottish LBC1936 (N=1001 unrelated)															
Moray House (IQ)	0.402	-0.03	0.03	**0.060**	0.06	0.03	***0.041***	0.07	0.03	0.766	-0.01	0.03	0.776	0.01	0.03
Matrix Reasoning	0.112	-0.05	0.03	0.603	0.02	0.03	0.980	0.001	0.03	0.762	-0.01	0.03	0.766	0.01	0.03
Letter Number Sequence	0.597	-0.02	0.03	0.763	0.01	0.03	0.134	0.05	0.03	0.383	0.03	0.03	0.442	-0.02	0.03
Dutch NTR (N=920 (340 families))															
Raven's Prog. Matrices	0.992	-0.001	0.06	0.281	-0.07	0.06	0.664	0.06	0.14	0.945	0.004	0.06	***$7.2x10^{-3}$***	0.22	0.08
Norwegian NCNG (N=670 unrelated)															
IQ	0.346	0.62	0.64	0.851	-0.12	0.64	0.554	1.03	1.74	**0.051**	1.15	0.59	0.943	0.05	0.70
Matrix Reasoning	0.229	0.29	0.24	***0.028***	0.52	0.24	0.282	-0.70	0.65	0.296	0.23	0.22	0.220	0.32	0.26
Letter Number Span	0.141	0.28	0.19	0.497	-0.13	0.19	**0.061**	-0.95	0.51	0.746	0.06	0.18	0.741	0.07	0.20
Digit Symbol	0.349	0.60	0.64	0.565	-0.36	0.63	0.359	-1.60	1.69	0.621	-0.29	0.59	0.289	-0.73	0.69

Combined Samples
Meta-analyses (IQ: N=7083 unrelated, Reasoning: N=6570 unrelated, Working Memory: N=1825 unrelated)

	**P value**	**z-score**	**P value**	**z-score**	**P value**	**z-score**	**P value**	**z-score**	**P value**	**z-score**
IQ[b]	***$9.0x10^{-3}$***	2.613	***0.033***	2.135	**0.057**	1.900	**0.082**	-1.739	***$1.1x10^{-3}$***	-3.264
Reasoning[c]	0.437	-0.777	0.798	0.256	0.756	0.310	0.729	0.346	***0.042***	2.030
Working Memory[d]	0.345	1.201	0.456	0.755	**0.096**	1.277	0.688	0.880	***0.026***	-1.503
	(0.230)	(0.945)	(0.450)	(0.745)	(0.202)	(1.664)	(0.379)	(0.402)	(0.133)	(-2.226)

NOTE: P values <0.05 are shown in bold and underlined, while those >0.05 but <0.10 are shown in bold only. At a gene-based level, in the Discovery sample, the genes *DGKB* and *ABHD4* were nominally associated with IQ (*p*=0.03, $8.1x10^{-4}$ respectively). *PRR5L* was not a VEGAS-listed gene. Traits are standardised (z-scores, M=0±1) for all cohorts excepting NCNG.

[a] The top-ranked loci for IQ was rs1242923.

[b]Meta-analysis for IQ included the following measures: Discovery - IQ (5 subtests of the Multidimensional Aptitude Battery), ALSPAC and NCNG - IQ (2 subtests of the WASI - includes Matrix Reasoning); LBC1936 - Moray House

[c]Meta-analysis for reasoning included the following measures: Discovery – RC ; ALSPAC/LBC1936/NCNG - Matrix Reasoning; NTR - Raven's Progressive Matrices

[d]Meta-analysis for working memory included the following measures: Discovery – Working Memory component; LBC1936 – Letter Number Sequence; NCNG – Digit Symbol (results using Letter Number Span are shown in brackets)
