# Supplementary material for: Genetic Basis of a Cognitive Complexity Metric
Source: PLoS One. 2015 Apr 10;10(4):e0123886. doi: 10.1371/journal.pone.0123886 (PMC4393228; doi:10.1371/journal.pone.0123886)
Supplement: S11 Table — (PDF) [file pone.0123886.s014.pdf]

Table S11. Gene Function.

**SNP**	**Gene**	**Location**	**Gene Function**
(Top ranked gene for IQ)	*FAM105A*	5p15.2	*FAM105A* (family with sequence similarity 105, member A): This is a proapoptotic gene not as yet well characterised [1].
rs7801010	*DGKB*	7p21.2	*DGKB* (diacylglycerol kinase, beta 90kDa): The diacylglycerol kinases have key roles in regulating many intracellular signalling proteins and are implicated in a range of human pathologies [2] including brain afflictions (e.g. *DGKH* with bipolar disorder and ***schizophrenia*** [3]). In addition, there are plausible links with cognitive function. Rat studies show that DGKB is an important modulator of protein kinase C [4], which is crucial for ***hippocampal memory formation*** [5]. Consistent with this finding, they also show DGKB involvement in dendritic spine shape and maturation in developing ***hippocampal neurons***, with flow-on effects for ***cognitive processes including memory*** [6,7]. Although little is known regarding the functional significance of multiple human *DGKB* isoforms, it has been suggested that altered relative levels of particular transcripts may influence emotional and ***cognitive behaviour*** by altering diacylglycerol turnover in the amygdala, caudate nucleus, and ***hippocampus*** [4]. More recently, *DGKB* was associated with fasting glucose homeostasis and type 2 diabetes in GWA meta-analyses [8] with a subsequent study finding it associated with insulin secretion [9]. Insulin has a profound effect on the brain, with insulin resistance underlying multiple chronic conditions known to impact ***cognitive function*** [10]. Gene-based tests in independent Norwegian (NCNG) and British (CAGES) samples, both of which contribute to the current association meta-analyses (Table S12), found suggestive evidence that the gene *DGKB* influences ***fluid intelligence*** ($p = 0.04$ and 0.001 respectively[11]). The current GWA meta-analyses suggested the minor allele of rs7801010 was associated with better cognitive ability.
rs2442756	*VPS13B*	8q22.2	*VPS13B* (vacuolar protein sorting 13 homolog B (yeast)) is a large multiexonic gene that shows alternative splicing. It has a broad expression pattern and is expressed differentially in the brain compared to other tissues (i.e. the major brain transcript (variant 1) is not the main form in other tissues [12]). It is proposed that alternative splicing may be of central importance for genes involved in information processing functions with the majority of alternative spliced genes found to be functionally involved in transmitting and regulating signals [13]. Mutations in the gene have been linked to Cohen syndrome [14], for which features include ***microcephaly (small head size)*** and moderate to severe ***intellectual impairment*** [15]. However, detailed gene function remains to be determined. In GWA meta-analyses conducted by the ENIGMA consortium (N=21,151) [16], the major allele of rs2442756 was associated with ***reduced hippocampal volume*** ($p = 0.018$) – for sample overlap see Table S12. The current GWA analyses suggested the major allele of rs2442756 was associated with worse relational processing ability in the Discovery sample, but the finding was not supported in the replication cohorts.
rs11195283	*RBM20*	10q25.2	Mutations in *RBM20* (RNA binding motif protein 20) have been associated with atrial fibrillation [17] and advanced disease in patients with dilated cardiomyopathy [18]. Atrial fibrillation is reported to be a determinant of low ***cognitive function*** in elderly men [19] and is associated with poorer ***cognitive outcomes*** in stroke patients [20,21]. The current GWA analyses suggested the minor allele of rs11195283 was associated with worse relational processing ability in the Discovery sample, but the finding was not supported in the replication cohorts.
rs4390263 (Plus: Top ranked gene for RC)	*NPS*	10q26.2	rs4390263 is 3.62 kb downstream of *NPS* (neuropeptide S) in a block of moderate linkage disequilibrium that extends from the beginning of the gene. NPS was first characterised in rodents as a modulator of sleep-wake cycles and anxiety [22]. With NPSR1 (neuropeptide S receptor 1), NPS forms a signalling system that has been implicated in susceptibility to multiple disorders in humans, including ***schizophrenia*** [23], panic disorder [24], and anxiety [25]. The NPSR1-NPS system is reported to modulate ***verbal memory*** consolidation in ***schizophrenia patients*** [23], consistent with a finding in mice whereby central NPS administration was able to dose dependently enhance ***memory retention*** [26]. In addition, it has been associated with activation levels in the dorsolateral prefrontal cortex (during the processing of fearful faces [24]), and in this capacity, it may also influence relational processing and working memory, which are known to engage this brain region [27,28]. In certain paradigms, it shows a pharmacological profile similar to clozapine (an atypical antipsychotic ***schizophrenia*** medication) and may be a potentially useful treatment for ***schizophrenia*** [29]. While results are as yet inconclusive, clozapine has been examined as a potential treatment of ***cognitive deficits associated with schizophrenia***, including verbal and visual learning, working memory, reasoning, and processing speed

			[see review 30]. In GWA meta-analyses by the Psychiatric Genomics Consortium (N=51,695) [31], rs4390263 was nominally associated with ***schizophrenia*** (p = 0.022), with the minor allele being protective. The current GWA meta-analyses suggested the minor allele was associated with better working memory performance.
rs12419146	*PRR5L*	11p13-p12	*PRR5L* (proline rich 5 like) is reported to play a role in regulating mRNA stability [32]. The current GWA meta-analyses suggested the minor allele of rs12419146 was associated with better cognitive ability.
rs1242923	*ABHD4*	14q11.2	Rodent studies suggest that *Abhd4* (abhydrolase domain containing 4) plays a role in the biosynthesis of endocannabinoids [33,34] and that endocannabinoid signalling is involved in ***learning and memory***. Multiple lines of evidence demonstrate that the system is involved in ***schizophrenia*** pathology (see review [35]). The current GWA meta-analyses suggested the minor allele of rs1242923 was associated with worse cognitive ability.
rs12882037	*ESRRB*	14q24.3	rs12882037 is 20.5 kb upstream of *ESRRB* (estrogen-related receptor beta) in a block of high linkage disequilibrium (0.8) that partly overlaps with the gene. Studies in mice suggest that Errb affects body composition, neuropeptide levels, stress hormones, and centrally-modulated startle responses [36]. Abnormal startle responses are found in ***schizophrenia*** patients [37] and have been investigated as an indicator of attention-dependent ***cognitive deficits*** [38,39]. The current GWA analyses suggested the minor allele of rs12882037 was associated with better relational processing ability in the Discovery sample, but the finding was not supported in the replication cohorts.
rs2837183	*DOPEY2*	21q22.2	*DOPEY2* (dopey family member 2) is a highly conserved gene containing leucine zipper-like domains with protein-protein interaction functions [40,41]. Studies suggest a conserved function in the control of morphogenesis (i.e. shapes of tissues, organs, entire organisms, and positions of the various specialised cell types), with a role in human morphogenesis of the cortex [40]. It is widely expressed in embryonic human CNS, but later in development, in the fetal brain, it becomes restricted to the cortex, cerebellum, and ***hippocampal formation*** – regions associated with ***learning and memory***, and regions where in Down syndrome, it is overexpressed, consistent with its location in the Down Syndrome Critical Region on chromosome 21 [42,43]. Thus, it is proposed as a candidate gene for a number of neurological alterations found in Down syndrome (i.e. ***hypoplasia of the hippocampus*** and cortex, smaller cerebellum, and ***mental retardation***). The current GWA analyses suggested the minor allele of rs2837183 was associated with worse relational processing ability in the Discovery sample, but the finding was not supported in the replication cohorts.

NOTE: Terms associated with cognitive function (including the hippocampus - a brain region commonly associated with memory function [44]) and with psychopathology are underlined and shown in bold with italcs.

References

1. Mannherz O, Mertens D, Hahn M, Lichter P (2006) Functional screening for proapoptotic genes by reverse transfection cell array technology. Genomics 87: 665-672.
2. Merida I, Avila-Flores A, Merino E (2008) Diacylglycerol kinases: at the hub of cell signalling. Biochem J 409: 1-18.
3. Zeng Z, Wang T, Li T, Li Y, Chen P, et al. (2011) Common SNPs and haplotypes in DGKH are associated with bipolar disorder and schizophrenia in the Chinese Han population. Mol Psychiatry 16: 473-475.
4. Caricasole A, Bettini E, Sala C, Roncarati R, Kobayashi N, et al. (2002) Molecular cloning and characterization of the human diacylglycerol kinase beta (DGKbeta) gene: alternative splicing generates DGKbeta isotypes with different properties. J Biol Chem 277: 4790-4796.
5. Dickstein DL, Weaver CM, Luebke JI, Hof PR (2013) Dendritic spine changes associated with normal aging. Neuroscience 251: 21-32.
6. Hozumi Y, Watanabe M, Otani K, Goto K (2009) Diacylglycerol kinase beta promotes dendritic outgrowth and spine maturation in developing hippocampal neurons. BMC Neurosci 10: 99.

7. Shirai Y, Kouzuki T, Kakefuda K, Moriguchi S, Oyagi A, et al. (2010) Essential role of neuron-enriched diacylglycerol kinase (DGK), DGKbeta in neurite spine formation, contributing to cognitive function. PLos ONE 5: e11602.
8. Dupuis J, Langenberg C, Prokopenko I, Saxena R, Soranzo N, et al. (2010) New genetic loci implicated in fasting glucose homeostasis and their impact on type 2 diabetes risk. Nat Genet 42: 105-116.
9. Ingelsson E, Langenberg C, Hivert MF, Prokopenko I, Lyssenko V, et al. (2010) Detailed physiologic characterization reveals diverse mechanisms for novel genetic Loci regulating glucose and insulin metabolism in humans. Diabetes 59: 1266-1275.
10. Cholerton B, Baker LD, Craft S (2013) Insulin, cognition, and dementia. Eur J Pharmacol 719: 170-179.
11. Christoforou A, Espeseth T, Davies G, Fernandes CPD, Giddaluru S, et al. (Epub ahead of print) GWAS-based pathway analysis differentiates between fluid and crystallized intelligence. Genes, Brain, and Behavior: doi: 10.1111/gbb.12152.
12. Velayos-Baeza A, Vettori A, Copley RR, Dobson-Stone C, Monaco AP (2004) Analysis of the human VPS13 gene family. Genomics 84: 536-549.
13. Lee CJ, Irizarry K (2003) Alternative splicing in the nervous system: an emerging source of diversity and regulation. Biol Psychiatry 54: 771-776.
14. Rivera-Brugues N, Albrecht B, Wieczorek D, Schmidt H, Keller T, et al. (2011) Cohen syndrome diagnosis using whole genome arrays. J Med Genet 48: 136-140.
15. Douzgou S, Petersen MB (2011) Clinical variability of genetic isolates of Cohen syndrome. Clin Genet 79: 501-506.
16. Stein JL, Medland SE, Vasquez AA, Hibar DP, Senstad RE, et al. (2012) Identification of common variants associated with human hippocampal and intracranial volumes. Nat Genet 44: 552-561.
17. Refaat MM, Lubitz SA, Makino S, Islam Z, Frangiskakis JM, et al. (2012) Genetic variation in the alternative splicing regulator RBM20 is associated with dilated cardiomyopathy. Heart Rhythm 9: 390-396.
18. Li D, Morales A, Gonzalez-Quintana J, Norton N, Siegfried JD, et al. (2010) Identification of novel mutations in RBM20 in patients with dilated cardiomyopathy. Clin Transl Sci 3: 90-97.
19. Kilander L, Andren B, Nyman H, Lind L, Boberg M, et al. (1998) Atrial fibrillation is an independent determinant of low cognitive function: a cross-sectional study in elderly men. Stroke 29: 1816-1820.
20. Kwok CS, Loke YK, Hale R, Potter JF, Myint PK (2011) Atrial fibrillation and incidence of dementia: a systematic review and meta-analysis. Neurology 76: 914-922.
21. Mizrahi EH, Waitzman A, Arad M, Adunsky A (2011) Atrial fibrillation predicts cognitive impairment in patients with ischemic stroke. Am J Alzheimers Dis Other Demen 26: 623-626.
22. Xu YL, Reinscheid RK, Huitron-Resendiz S, Clark SD, Wang Z, et al. (2004) Neuropeptide S: a neuropeptide promoting arousal and anxiolytic-like effects. Neuron 43: 487-497.
23. Lennertz L, Quednow BB, Schuhmacher A, Petrovsky N, Frommann I, et al. (2012) The functional coding variant Asn107Ile of the neuropeptide S receptor gene (NPSR1) is associated with schizophrenia and modulates verbal memory and the acoustic startle response. Int J Neuropsychopharmacol 15: 1205-1215.
24. Domschke K, Reif A, Weber H, Richter J, Hohoff C, et al. (2011) Neuropeptide S receptor gene -- converging evidence for a role in panic disorder. Mol Psychiatry 16: 938-948.
25. Donner J, Haapakoski R, Ezer S, Melen E, Pirkola S, et al. (2010) Assessment of the neuropeptide S system in anxiety disorders. Biol Psychiatry 68: 474-483.

26. Okamura N, Garau C, Duangdao DM, Clark SD, Jungling K, et al. (2011) Neuropeptide S enhances memory during the consolidation phase and interacts with noradrenergic systems in the brain. Neuropsychopharmacology 36: 744-752.
27. D'Esposito M (2007) From cognitive to neural models of working memory. Philos Trans R Soc Lond B Biol Sci 362: 761-772.
28. Krawczyk DC, Michelle McClelland M, Donovan CM (2011) A hierarchy for relational reasoning in the prefrontal cortex. Cortex 47: 588-597.
29. Okamura N, Reinscheid RK, Ohgake S, Iyo M, Hashimoto K (2010) Neuropeptide S attenuates neuropathological, neurochemical and behavioral changes induced by the NMDA receptor antagonist MK-801. Neuropharmacology 58: 166-172.
30. Vingerhoets WA, Bloemen OJ, Bakker G, van Amelsvoort TA (2013) Pharmacological Interventions for the MATRICS Cognitive Domains in Schizophrenia: What's the Evidence? Front Psychiatry 4: 157.
31. The_Schizophrenia_Psychiatric_Genome-Wide_Association_Study_(GWAS)_Consortium (2011) Genome-wide association study identifies five new schizophrenia loci. Nat Genet 43: 969-976.
32. Holmes B, Artinian N, Anderson L, Martin J, Masri J, et al. (2012) Protor-2 interacts with tristetraprolin to regulate mRNA stability during stress. Cell Signal 24: 309-315.
33. Liu J, Wang L, Harvey-White J, Huang BX, Kim HY, et al. (2008) Multiple pathways involved in the biosynthesis of anandamide. Neuropharmacology 54: 1-7.
34. Simon GM, Cravatt BF (2006) Endocannabinoid biosynthesis proceeding through glycerophospho-N-acyl ethanolamine and a role for alpha/beta-hydrolase 4 in this pathway. J Biol Chem 281: 26465-26472.
35. Saito A, Ballinger MD, Pletnikov MV, Wong DF, Kamiya A (2013) Endocannabinoid system: potential novel targets for treatment of schizophrenia. Neurobiol Dis 53: 10-17.
36. Byerly MS, Swanson RD, Wong GW, Blackshaw S (2013) Estrogen-related receptor beta deficiency alters body composition and response to restraint stress. BMC Physiol 13: 10.
37. Geyer MA, Swerdlow NR, Mansbach RS, Braff DL (1990) Startle response models of sensorimotor gating and habituation deficits in schizophrenia. Brain Res Bull 25: 485-498.
38. Braff DL, Light GA (2004) Preattentional and attentional cognitive deficits as targets for treating schizophrenia. Psychopharmacology (Berl) 174: 75-85.
39. Kishi T, Fukuo Y, Okochi T, Kawashima K, Moriwaki M, et al. (2012) The relationship between acoustic startle response measures and cognitive functions in Japanese patients with schizophrenia. Neuromolecular Med 14: 131-138.
40. Rachidi M, Lopes C, Costantine M, Delabar JM (2005) C21orf5, a new member of Dopey family involved in morphogenesis, could participate in neurological alterations and mental retardation in Down syndrome. DNA Res 12: 203-210.
41. Pascon RC, Miller BL (2000) Morphogenesis in Aspergillus nidulans requires Dopey (DopA), a member of a novel family of leucine zipper-like proteins conserved from yeast to humans. Mol Microbiol 36: 1250-1264.
42. Rachidi M, Delezoide AL, Delabar JM, Lopes C (2009) A quantitative assessment of gene expression (QAGE) reveals differential overexpression of DOPEY2, a candidate gene for mental retardation, in Down syndrome brain regions. Int J Dev Neurosci 27: 393-398.
43. Rachidi M, Lopes C, Delezoide AL, Delabar JM (2006) C21orf5, a human candidate gene for brain abnormalities and mental retardation in Down syndrome. Cytogenet Genome Res 112: 16-22.
44. Kitamura T, Inokuchi K (2014) Role of adult neurogenesis in hippocampal-cortical memory consolidation. Mol Brain 7: 13.
