# Supplementary material for: Genetic Basis of a Cognitive Complexity Metric
Source: PLoS One. 2015 Apr 10;10(4):e0123886. doi: 10.1371/journal.pone.0123886 (PMC4393228; doi:10.1371/journal.pone.0123886)
Supplement: S12 Table — (PDF) [file pone.0123886.s015.pdf]

Table S12. Sample Overlap Between Current Association and Previous Genome-Wide Association Meta-Analyses of Cognitive and Related Phenotypes

	Number of Participants				
	Adult Cognition [1,2]	Childhood Cognition [3]	Educational Attainment [4]	Hippocampal Volume [5]	Schizophrenia [6]
Discovery Sample	-	1725	-	485	-
ALSPAC[a]	-	4078	-	-	-
LBC1936	1005	947	1005	249	-
NTR	-	739[b]	183	-	-
NCNG	670	-	-	327	-

[a]Parental data contributed to Educational Attainment
[b]Replication sample.

References

1. Davies G, Tenesa A, Payton A, Yang J, Harris SE, et al. (2011) Genome-wide association studies establish that human intelligence is highly heritable and polygenic. Mol Psychiatry 16: 996-1005.
2. Christoforou A, Espeseth T, Davies G, Fernandes CPD, Giddaluru S, et al. (Epub ahead of print) GWAS-based pathway analysis differentiates between fluid and crystallized intelligence. Genes, Brain, and Behavior: doi: 10.1111/gbb.12152.
3. Benyamin B, Pourcain B, Davis OS, Davies G, Hansell NK, et al. (2013) Childhood intelligence is heritable, highly polygenic and associated with FNBP1L. Mol Psychiatry 19: 253-258.
4. Rietveld CA, Medland SE, Derringer J, Yang J, Esko T, et al. (2013) GWAS of 126,559 individuals identifies genetic variants associated with educational attainment. Science 340: 1467-1471.
5. Stein JL, Medland SE, Vasquez AA, Hibar DP, Senstad RE, et al. (2012) Identification of common variants associated with human hippocampal and intracranial volumes. Nat Genet 44: 552-561.
6. The_Schizophrenia_Psychiatric_Genome-Wide_Association_Study_(GWAS)_Consortium (2011) Genome-wide association study identifies five new schizophrenia loci. Nat Genet 43: 969-976.
