# Supplementary material for: Genetic Basis of a Cognitive Complexity Metric
Source: PLoS One. 2015 Apr 10;10(4):e0123886. doi: 10.1371/journal.pone.0123886 (PMC4393228; doi:10.1371/journal.pone.0123886)
Supplement: S1 Text — (PDF) [file pone.0123886.s016.pdf]

Text S1. Relational Complexity Tasks: each contained items at two (ternary- and quaternary-relational) or three (binary- ternary- and quaternary-relational) levels of complexity and assessed relational processing in different content domains [1]. Computer presentation of each of the tasks provided both instruction and automatic timing and scoring, and ensured task conformity. For each task the main dependent measure was accuracy and no time limits were imposed.

(A) The **Sentence Comprehension** task required processing of the noun-verb relations. It was adapted from Andrews et al. [1] and comprised a total of 22 sentences containing relative clauses. There were Subject-relative and Object-relative sentences, each requiring either 3 or 4 role assignments. Sentences were presented one at a time on the screen. Participants were asked to read each sentence carefully and when they understood the sentence, to move to a probe question (at which time the sentence could no longer be seen) and select their answer from the options available. The probe question assessed comprehension by asking about a noun-verb relation, such as “who (action specified)?”, or “what did (specified person) do?”. Of the 22 sentences, six were subject relative and 16 object-relative with 50% of sentences requiring processing of 3-role noun-verb relationships and 50% requiring 4-role assignments. These 4 sentence types (subject, object relative; 3-, 4-role) were randomized and presented in the same order for each participant. Instructions were given on the screen, with the opportunity to address questions to a research assistant if necessary. The task was scored by summing the number of correct responses, with a maximum score of 22.

(B) The **N-term** task is an extended version of a transitive inference task and was adapted from Andrews et al. [1]. In this task, relationships are inferred from the premises provided and thus inferential reasoning is required. In the 4-term example, the premises consist of a set of 4 paired letters, with the relationship between the letters indicated by a combination of greater than (>) and less than (<) signs (e.g. B > A) Participants are instructed to order the letters from greatest to smallest according to the information in the premises, and to construct the entire sequence mentally before beginning to type the sequence into the boxes. Once a letter has been entered participants are unable to reorder them. The premise information contained two levels of complexity: 3-term and 4-term items, with 8 trials presented for each complexity level using a blocked design. Each block was preceded by an example and one practice item, after which, participants had the opportunity to ask questions if anything was unclear. In scoring responses as correct or incorrect, relationship rather than direction was taken as the key element. Thus where participants confused the “<” and “>” signs, answers that were in the reverse of the correct order (i.e. that were ordered from smallest to greatest) were deemed to be correct. The task was scored by summing the number of correct sequences, with a maximum score of 16.

(C) The **Latin Square** task was adapted from Birney et al. [2,3]. It comprises a ‘problem square’ consisting of a 4 × 4 matrix, with each cell either containing one of four shapes or no shape (i.e. remaining empty). The task was to determine which shape, selected from an ‘option’ panel, should occur in a specified cell (indicated by a question mark). In the ‘completed square’, each shape should occur only once in each row and each column. Three practice trials of increasing complexity were presented, with a research assistant explaining and guiding the participant on how the correct response is determined. Participants were instructed to work through the problems as quickly as possible without sacrificing accuracy, and to do all the work in their heads. A total of 12 problems divided equally between 3 complexity levels (binary-relational, ternary-relational, and quaternary-relational) were then presented.

References

1. Andrews G, Birney D, Halford GS (2006) Relational processing and working memory capacity in comprehension of relative clause sentences. Mem Cognit 34: 1325-1340.
2. Birney DP, Halford GS, Andrews G (2006) Measuring the influence of complexity on relational reasoning. Educational and Psychological Measurement 66: 146-171.
3. Birney DP, Bowman DB, Beckmann J, Seah Y (2012) Assessment of processing capacity: Latin-square task performance in a population of managers. European Journal of Psychological Assessment 28: 216-226.
