# Supplementary material for: Genetic Basis of a Cognitive Complexity Metric
Source: PLoS One. 2015 Apr 10;10(4):e0123886. doi: 10.1371/journal.pone.0123886 (PMC4393228; doi:10.1371/journal.pone.0123886)
Supplement: S2 Text — (PDF) [file pone.0123886.s017.pdf]

Text S2. Structural Equation Modelling. Figures 2-3 show variance and covariance partitioned into additive genetic (A) and non-shared environmental (E) sources of variance.

Figure 2 (common pathway model [1]) shows that genetic sources accounted for 41-57% of the variance in the individual tasks (i.e. heritability (h^2) ranged 0.41-0.57). The latent relational processing factor was highly heritable (86%) and accounted for all of the genetic variance in the N-term Task (i.e. (0.93^2x0.79^2)/0.54), 72% for the Sentence Task, and 69% for Latin Square. In contrast, unique environmental influences were mainly specific to each variable with specific influences (E_S) accounting for 83-92% of total environmental variance (e.g. Sentence Task = 37/(100-57). This model confirms a strong latent factor and shows that it is largely genetic in nature. Heritability was maximised as the factor is free of uncorrelated measurement error, which is partitioned into the specific environmental (E_S) pathways.

Figure 3 (Cholesky decomposition [1]) partitioned covariation between RC, IQ, Reasoning and Working Memory into additive genetic (A) and non-shared environmental sources (E). The genetic source influencing RC (A_1) accounted for 59% of genetic variance in IQ (50/85), 69% for Reasoning (44/64), and 39% for Working Memory (25/64). For more detail regarding covariation between RC and IQ see Figure S2. Genetic sources (A_1, A_2, A_3) accounted for 89% of covariation between the traits Reasoning and Working Memory (r_p=0.52) (using tracing rules of path analysis [2,3]: ((0.67x0.50)+(0.30x0.16)+(0.32x0.25))/0.52), with unique environment accounting for the remaining 11%. Further, the covariation between Reasoning and Working Memory was largely influenced by sources also influencing RC. These accounted for 67% of the total (((0.67x0.50)+(0.11x0.10))/0.52) and 72% of the genetic covariation ((0.67x0.50)/((0.67x0.50) +(0.30x0.16)+(0.32x0.25))). Alternatively, of the covariation associated with RC, 97% was genetic. Independent of RC, IQ accounted for 12% of the total covariation (((0.30x0.16)+(0.21x0.07))/0.52) between Reasoning and Working memory and 10% of the genetic covariation ((0.30x0.16)/((0.67x0.50)+(0.30x0.16)+(0.32x0.25))). Note that due to the substantial overlap between RC and IQ, if IQ is allowed priority, it accounts for 75% of the genetic covariation between Reasoning and Working Memory, while RC independently accounts for a further 8%. All of the above multivariate analyses allowed for only A and E sources of influence as common environmental sources could be dropped at the univariate level (Table S4).

References

1. Neale MC, Cardon LR (1992) Methodology for genetic studies of twins and families. Dordrecht: Kluwer Academic Publishers.
2. Wright S (1918) On the nature of size factors. Genetics 3: 367-374.
3. Wright S (1934) The method of path coefficients. Annals of Mathematical Statistics 5: 161-215.
